# A data driven methodology for social science research with left-behind children as a case study

**DOI:** 10.1371/journal.pone.0242483

**Published:** 2020-11-20

**Authors:** Chao Wu, Guolong Wang, Simon Hu, Yue Liu, Hong Mi, Ye Zhou, Yi-ke Guo, Tongtong Song

**Affiliations:** 1 School of Public Affairs, Zhejiang University, Hangzhou, Zhejiang, China; 2 School of Civil and Environmental Engineering, ZJU-UIUC Institute, Zhejiang University, Haining, China; 3 Data Science Institute, Imperial College London, London, United Kingdom; 4 College of Software Technology, Zhejiang University, Hangzhou, Zhejiang, China; Ningbo University, China, UNITED STATES

## Abstract

For decades, traditional correlation analysis and regression models have been used in social science research. However, the development of machine learning algorithms makes it possible to apply machine learning techniques for social science research and social issues, which may outperform standard regression methods in some cases. Under the circumstances, this article proposes a methodological workflow for data analysis by machine learning techniques that have the possibility to be widely applied in social issues. Specifically, the workflow tries to uncover the natural mechanisms behind the social issues through a data-driven perspective from feature selection to model building. The advantage of data-driven techniques in feature selection is that the workflow can be built without so much restriction of related knowledge and theory in social science. The advantage of using machine learning techniques in modelling is to uncover non-linear and complex relationships behind social issues. The main purpose of our methodological workflow is to find important fields relevant to the target and provide appropriate predictions. However, to explain the result still needs theory and knowledge from social science. In this paper, we trained a methodological workflow with left-behind children as the social issue case, and all steps and full results are included.

## 1 Introduction

Traditional data analysis methodology has encountered some challenges in understanding the underlying complex mechanism of social issues. There are two main challenges. First, with the growing data volume, it becomes harder to quickly select features manually based on prior knowledge, domain expert experience, and literature review. Second, traditional methods cannot capture complex non-linear relationships underlying the explanatory variables and target variables as they usually assume a linear relationship between variables. So even with large datasets, traditional data analysis methods have difficulty in making full use of the rich information timely compared with machine learning techniques.

First, feature selection is a process of choosing a subset of original features so that the feature space is optimally reduced [1], which is believed to be an essential data processing step prior to the application of methods and algorithms [1–3]. There are several goals for feature selection, such as to avoid overfitting and improve model performance [4], to provide faster and more cost-effective models without loss of prediction accuracy [1] and to uncover a more comprehensive and deeper nature of the processed data and learned results [4–6]. However, in traditional social science research, feature selection criteria are usually based on related literature review, prior experience, expert interviews such as Delphi Method [7], and the limitations of data sets and methods. Some limits about such criteria are shown below. First, what if the prior knowledge does not uncover the natural mechanism of social issues sufficiently as the world is consistently changing. Second, a large volume of data has been created so that large scale features can be selected in the era of big data. Third, the fast development and application of feature selection techniques in machine learning might move forward the research boundary if we do not hold prior principles strictly. Feature selection techniques such as filter approach, wrapper approach, embedded approach, and hybrid approach [4, 8] have been developed in the field of machine learning, bioinformatics, and data mining. So we introduce feature selection techniques of machine learning in our methodology workflow, specifically filter approach and wrapper approach in our left-behind children case study.

Second, traditional methods in social science research such as correlation and traditional regression analysis [9] are usually used to explore dynamically changing social phenomenon. Correlation usually interprets the strength of linear associations between two variables, while traditional regression analysis provides an evaluation of the impact of predictor variables on dependent variables [10]. Indeed, traditional methods provide a means to understand social issues. However, there are some problems with these methods. First, traditional regression methods usually assume a linear relationship between variables, which does not reflect the true relationship of dynamically changing variables. Second, it is increasingly difficult to accurately predict what will happen and reasonably explain why to the decision-makers by linear or logistic regression models as they cannot capture complex mechanisms.

Furthermore, previous researches have made sufficient comparison on machine learning and traditional regression methods. Research on predicting house values indicates that Neural Network Regression (NNR) shows better predictive performance than multiple regression analysis in a moderate-to-large size data sample. Besides, the predictive performance of the neural network increases as the data sample size increases in different functional model specification [11]. Research on predicting electric power also proves that neural network outperforms statistical technique in forecasting [12]. Research about predictive models for hepatocellular carcinoma (HCC) indicates that machine learning algorithms had significantly better diagnostic accuracy than conventional regression models. Machine learning algorithms offer a novel methodology, which may improve HCC risk prognostication among patients with cirrhosis. Moreover, the machine learning models are capable of catching sophisticated non-linear integrating effects [13]. An analysis of predicting seed yield of safflower shows that predicting by neural networks leads to more accurate results than linear models, due to the account of non-linearity [14]. Research even showed that a non-optimized neural network model can also outperform multiple regression analysis in modeling bridge risks [15]. An important comparison between neural network models and logistic regression shows that the main advantage of NNR models lies in their hidden layers of nodes. NNR is particularly useful when there are implicit interactions and complex relationships in data sets [16]. Some research compared Support Vector Regression (SVR), which also has the ability to catch the non-linearly relationship, with multiple regression analysis and NNR. SVR performs better than linear regression [17], while the difference in prediction performance of SVR and NNR does not reach a conclusion in papers about stock index prediction [17, 18]. To sum up, it can be known clearly that there is a high probability that a better prediction result and a better description of non-linear relationships can be expected by applying machine learning techniques instead of traditional regression.

Based on the above, this paper proposes a general data-driven methodological workflow, which can be widely used for different social issues, to overcome the weakness of traditional methods on feature selection and capture the non-linearity using machine learning techniques and methods. There are two main innovations in our workflow. First, we introduce the Filter and Wrapper Approach to select features from large data sets. Second, we introduce machine learning methods such as NNR and SVR to social science research rather than their traditional application in natural science or engineering problems to uncover complex non-linear relationships. We also tried several other deep models but found that NNR and SVR have better performance on both accuracy and overfitting. And NNR and SVR are much more simple and computationally efficient than other deep models.

The proposed workflow can act as a tool for researchers to conduct research in unfamiliar fields, because we no longer require prior knowledge to select features. The implementation of our data-driven methodology may not only promote the development of interdisciplinary research but also provide an in-depth understanding of current empirical research on social issues. The weakness of applying machine learning techniques is that the result may lack a comprehensive explanation. However, the characteristics such as uncovering the non-linear and complexity of the phenomenon, providing a wide range of applications, and creating an automatic process from feature selection to modelling are all advantages that promote us to apply them to social science research.

This paper takes left-behind children as a case study to form the methodological workflow, which contains feature selection and modelling, assessment of the model, and voting for key fields in Chapter 2 Method. Then we analyze the performance of our proposed methodological workflow through scenario simulation and conclusion of the case study in Chapter 3 Result. Afterward, we discuss the methodology, get a conclusion, and refer to future work about our proposed methodology in Chapters 4, 5, and 6.

## 2 Method

In this section, we provide an overview of the proposed methodology and explain each step in-detail on how it helps researchers on modelling as well as predicting future trends based on historical data. Our method will be shown in the case of the left-behind children issue study. The introduction of left-behind children issue study can be found in the S1 Appendix: Case Study.

### 2.1 Overview

**Fig 1:**
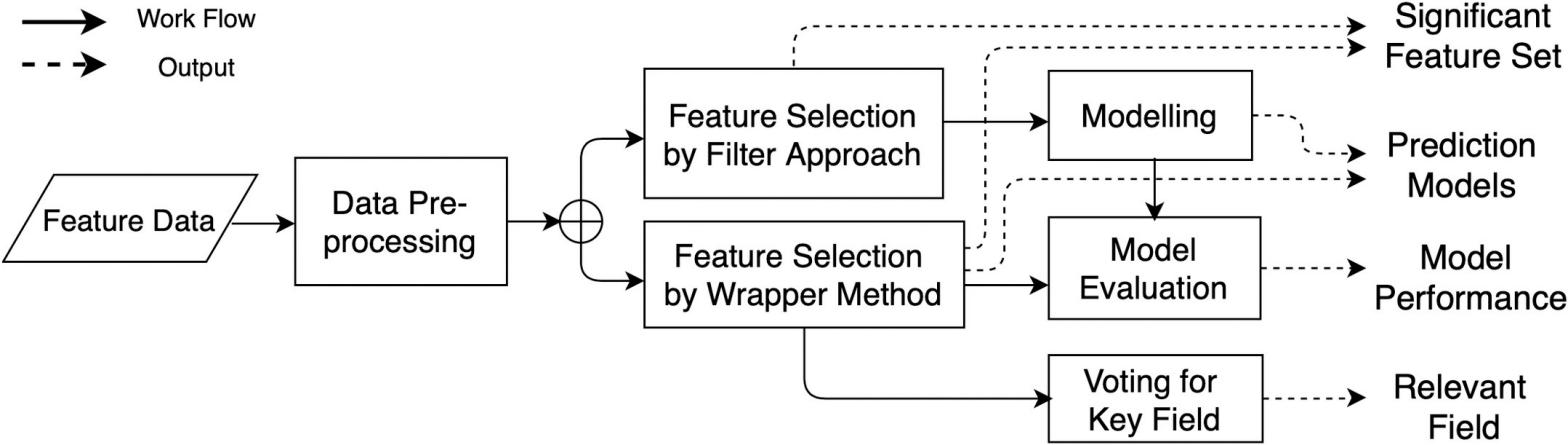
Workflow design.

The main steps of our methodological workflow are presented in Fig 1. The blocks and solid arrows represent the sequence of the workflow and dashed arrows give the results obtained from each block. The key steps are as follows: First, the raw data is pre-processed into a standard form for subsequent steps. Then, we use both Filter and Wrapper Approaches to select features. The Filter Approach selects features independently, however, the Wrapper Approach is implemented with modelling procedure as shown in Fig 1. After Filter Approach and during Wrapper Approach feature selection, SVR and NNR are applied in modelling, prediction, and model comparisons. Finally, since we can get many feature combinations from Wrapper Approach that perform well in modelling, we design the “Voting for key fields” (that will be denoted as VKF) procedure to find which field is more statistically related to the target.

### 2.2 Pre-processing

**Fig 2:**
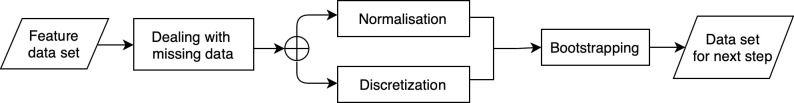
Pre-processing.

Before selecting features with the Filter and Wrapper Approach, we should pre-process the data first. Two data sets–Chinese Migration Data and Province-level Data are used to form the proposed workflow in our left-behind children case study. The introduction of the two data sets and the detailed process of pre-processing can be found in the S1 Appendix: Data. As shown in the Appendix part, the raw data are unable to be used directly as the input of feature selection and modelling because of missing values, datatype differences, unbalanced values, etc. Hence, a pre-processing [19] step is required, such as data deletion and imputation for missing data [20], discretization, normalization, and bootstrapping [21] that showed in Fig 2.

First, we should deal with missing data. Missing values or incomplete data is unavoidable in most social issues data sources. There are several methods on how to deal with the missing values, such as data deletion, mean substitution [22], hot-deck imputation [23], regression-based, and a propensity score classifier [24].

Then, normalization and discretization should be applied to our dataset. Normalization is a feature scaling transformation method when there is a large difference between the maximum and minimum value of a feature. There are several methods for normalization, such as min-max normalization and z-score normalization. We use the previous one.

And data discretization occurs when it involves transferring continuous variables to discrete ones. There are several methods for discretization, such as error-based method and entropy-based method. In our case, we divided the interval into K partitions and calculated the maximum and the minimum of the features that deserved to be discretized so that partitions of the range were observed into K equal-sized intervals [19]. Also, the 2K rule is used to determine the number of classes [25].

Finally, bootstrapping is a general resampling procedure for estimating the distributions of statistics based on independent observations [21]. There are several methods of bootstrapping, such as parametric bootstrap and non-parametric bootstrap [26]. In this paper, we introduce the bootstrapping method to overcome the lack of data examples, and we add some Gaussian noises on the original data to obtain new samples. It was conducted with several choices of standard deviation, and we select one to avoid overfitting through experiments.

### 2.3 Feature selection and model evaluation

**Fig 3:**
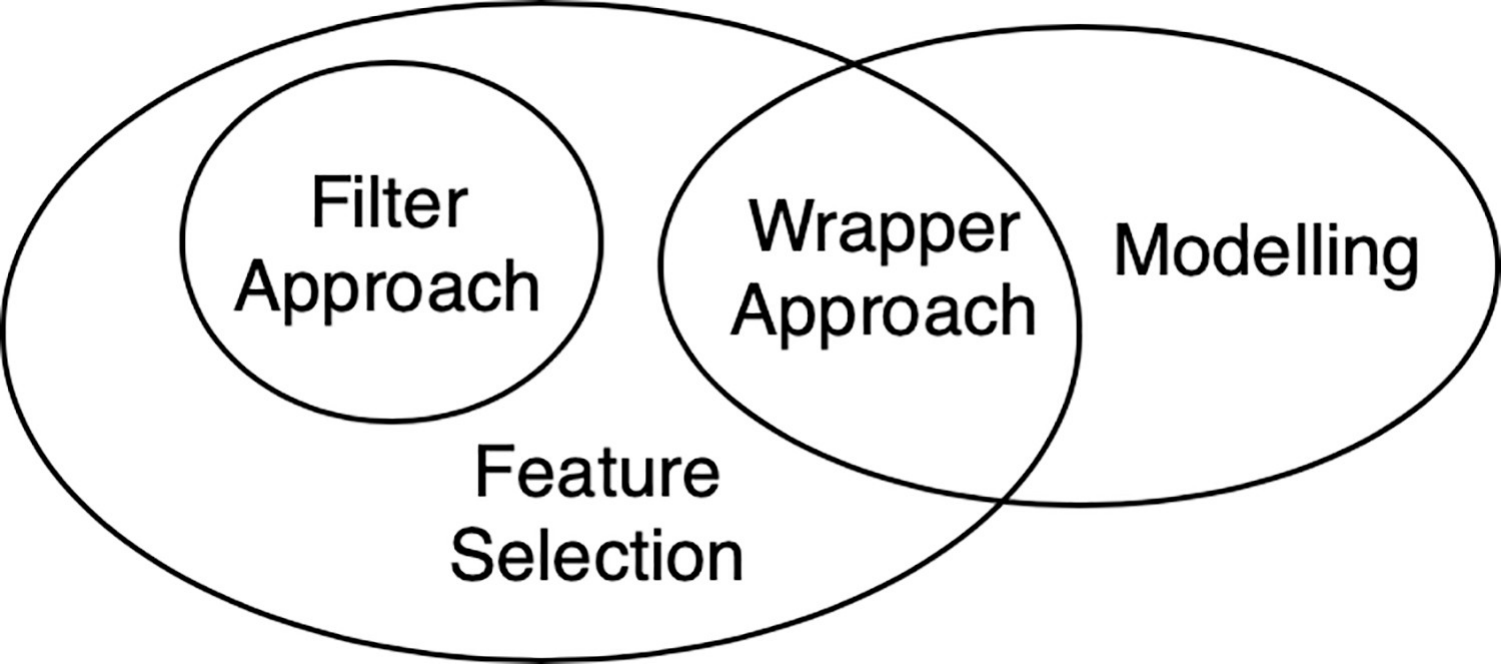
The relationship for feature selection and modelling.

After pre-processing, we move forward to feature selection and modelling, which is an essential part of our methodological workflow. In our left-behind children case, for feature selection, we use Filter Approach and Wrapper Approach. For modelling, we use SVR and NNR models. The relationship between feature selection and modelling in our work is presented in Fig 3. The modelling procedure is accomplished after the application of the Filter Approach and during Wrapper Approach, respectively.

**Fig 4:**
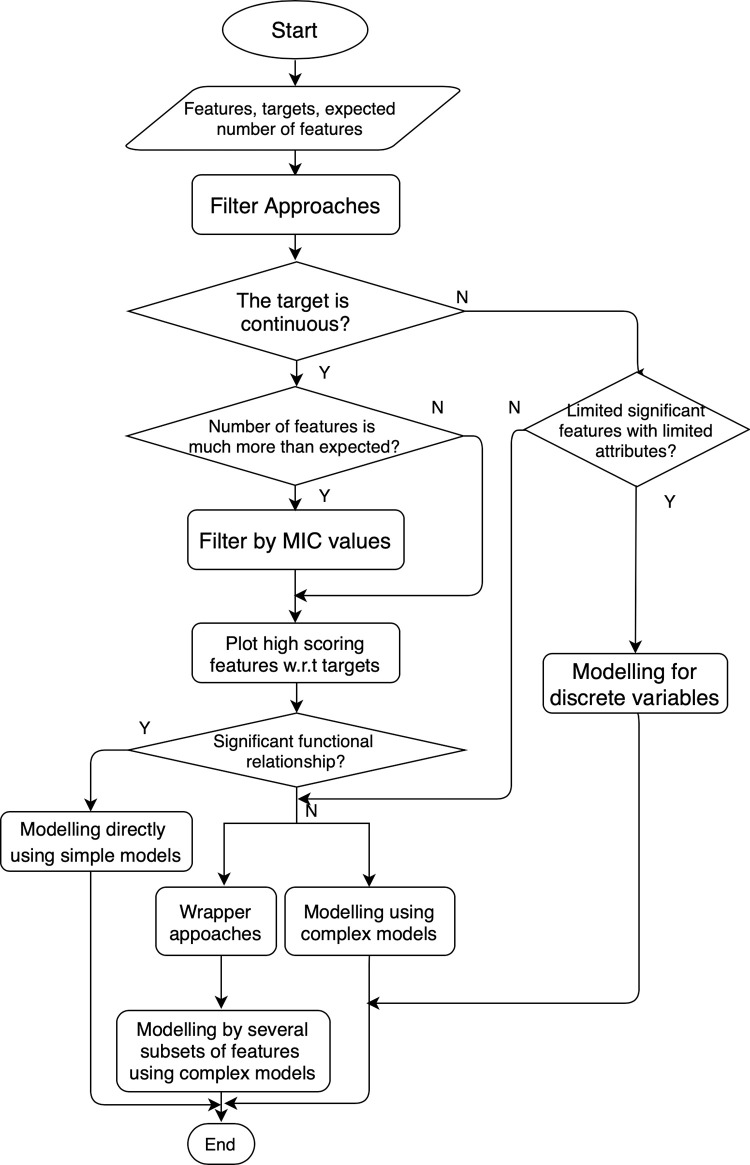
Feature selection and modelling.

Filter Approach selects feature subsets on the basis of intrinsic characteristics of the data, independent of the mining algorithm [7]. While Wrapper Approach requires a predetermined algorithm, which in our case is the regression model, to determine the best feature subset and the predictive accuracy of the model is used for evaluation [5]. The Wrapper Approach guarantees better results as it uses predictive accuracy to evaluate, but it is computationally expensive for large data sets. So in our workflow, Filter Approach should always be used first since it has the advantage of computationally simple, fast [4], and rapidly scalable [27]. If some significant relationships are discovered through Filter Approach, there is no need for further complex methods. But if the results are not as good as expected, then the Wrapper Approach should be implemented. The flow chart for the interrelationship of feature selection and modelling is given in Fig 4.

#### 2.3.1 Filter approach and modelling

*Feature selection*. Filter Approach is used to analyze correlations. Many measures are proposed to judge the correlation between two variables by rating with a score. The Filter Approach is used for both data sets in our left-behind children case study.

1 Chinese Migration Data

The features of Chinese Migration Data are listed in Table A in S1 Appendix and the label is isLeftBehind as defined. All data have been processed into discrete variables. According to Fig 4 and because of discrete values, the L1 metric and Information Entropy are used to measure the relationship between each feature and target. The measures are calculated and then ranked. Some of the higher scoring features are shown in Table 1.

**Table 1:** High scoring features.

Measure Rank	1	2	3	4	5	6	7
**Entropy**	marriage	hukou	from	industry	job	Edu	company
**L1 Metric**	marriage	edu	range	from	hukou	industry	job

Generally speaking, marriage, hukou (the property of registered permanent residence, rural or urban), edu, and from score high for both measures, which are more relevant to the target (whether a child is left-behind or not). The features edu and from, which are not used to mark the isLeftBehind label, scores close to other features implying a strong correlation.

2 Province-level Data

The features of Province-level Data are listed in Table B in S1 Appendix and the target is the incidence of left-behind children. Unlike the data in Chinese Migration Data, the features and target variables are continuous. According to Fig 4, Pearson’s Correlation Coefficient (CC) [28], Coefficient of Determination (*r*^*2*^) [29], and Maximum Information Coefficient (MIC) are used as the measures. The measures between the target and each feature are calculated and then ranked. The high scoring features are listed in Tables 2–4 respectively.

**Table 2:** Top 10 high scoring features for CC measure.

	Filtered Feature	CC
**1**	museum visitors	0.7302
**2**	highway passenger traffic	0.7155
**3**	secondary vocational school graduates eligible for qualifications	0.7072
**4**	special education enrollment	0.7032
**5**	national special entitled groups with regular subsidy	0.7021
**6**	passenger volume	0.6977
**7**	national special entitled groups	0.6908
**8**	construction employments in urban units	0.6819
**9**	student-teacher ratio of primary school	0.6745
**10**	rural population	0.6694

**Table 3:** Top 10 high scoring features for MIC measure.

	Filtered Feature	MIC
**1**	special education enrollment	0.8215
**2**	vegetable acreage	0.8215
**3**	number of special education students	0.8215
**4**	national special entitled groups with regular subsidy	0.7412
**5**	national special entitled groups	0.7412
**6**	office sales	0.7320
**7**	number of health checked at outpatient	0.7070
**8**	secondary vocational school enrollment	0.6969
**9**	number of secondary vocational school students	0.6969
**10**	secondary vocational school graduates eligible for qualifications	0.6969

**Table 4:** Top 10 high scoring features for r^2^ measure.

	Filtered Feature	r^2^
**1**	highway passenger traffic	0.3323
**2**	passenger volume	0.3269
**3**	student-teacher ratio of primary school	0.3085
**4**	number of inpatients	0.2941
**5**	number of discharged patients	0.2928
**6**	health expenditure from local finance	0.2869
**7**	number of high schools	0.2787
**8**	secondary vocational school graduates eligible for qualifications	0.2262
**9**	construction area of residential house	0.2162
**10**	number of automatic weather stations	0.2104

As shown above, in Tables 2–4, feature score ranking varies due to different measurement methods, but education-related features appear in the top 10 lists frequently. According to the values of measures, the top-scoring features are selected. We found that the plots of the features with respect to the target do not show a strong functional relationship. Therefore, multiple features will be used in the modelling part.

*Modelling*. Our models are built to predict the incidence of left-behind children on the province level, so the features selected from Province-level Data, which are shown in Tables 2–4 are used to build SVR and NNR models for prediction. For each kind of model, the top 5 and 10 features are used to do modelling respectively. Meanwhile, three different measures are used for each model. Therefore, 12 models in total are built using the features selected by the Filter Approach. The parameters of the two regression models are listed in Tables 5 and 6.

**Table 5:** Parameters for SVR model with filtered features.

Trade-off Constant C	Kernal Function	*γ*
1000	RBF	0.1

**Table 6:** Parameters for NNR model with filtered features.

Number of Hidden Neurons		Loss Function	Learning Algorithm	Learning Rate	Coefficient of L2 Regularization	Activation Function
5 Inputs	10 Inputs	MSE	SGD	0.01	0.01	ReLU, Tanh
5	8					

The 12 models’ performance is listed below in Tables 7 and 8, with the combination of three measurements, two feature numbers, and two models.

**Table 7:** Performances for SVR models built by filter approach.

Feature selection methods		Performance	
Number of features	Filter measure	*r*^2^	MSE
**5 features**	CC	0.4717	0.0492
	MIC	0.5668	0.0404
	*r*^2^	0.5788	0.0392
**10 features**	CC	0.7054	0.0274
	MIC	0.4552	0.0508
	*r*^2^	0.6715	0.0306

**Table 8:** Average performance for NNR models built by filter approach.

Feature selection methods		Performance	
Number of features	Filter measure	*r*^2^	MSE
**5 features**	CC	0.4334	0.0528
	MIC	0.3796	0.0578
	*r*^2^	0.3912	0.0567
**10 features**	CC	0.5616	0.0408
	MIC	0.4360	0.0524
	*r*^2^	0.5274	0.0440

1 Support Vector Regression

As shown in Table 7, the performance value of *r*^2^-measurement increases when the number of features doubled, while the MIC’s decreases. For SVR models, the results imply that a larger set of features doesn’t necessarily mean better performance and that different combinations of features may influence the performance of the models.

2 Neural Network Regression

For NNR models, an average of 20 repeated experiments is used to evaluate its performance. According to Table 8, the models built with the features selected by the Correlation Coefficient measure perform better than the others. In addition, the results show that more features for the NNR model do mean better performance, to some extent.

According to the results in Tables 7 and 8, SVR models give higher *r*^2^-performance values than NNR models, which means SVR models have stronger ability of prediction. In addition, the training time of the SVR model is shorter than NNR model, since there are fast algorithms implemented for solving quadratic programming problems. Therefore, for the Filter Approach, SVR is better than NNR most of the time in our case. Then we choose from SVR models above to predict the province-level incidence of left-behind children.

**Fig 5:**
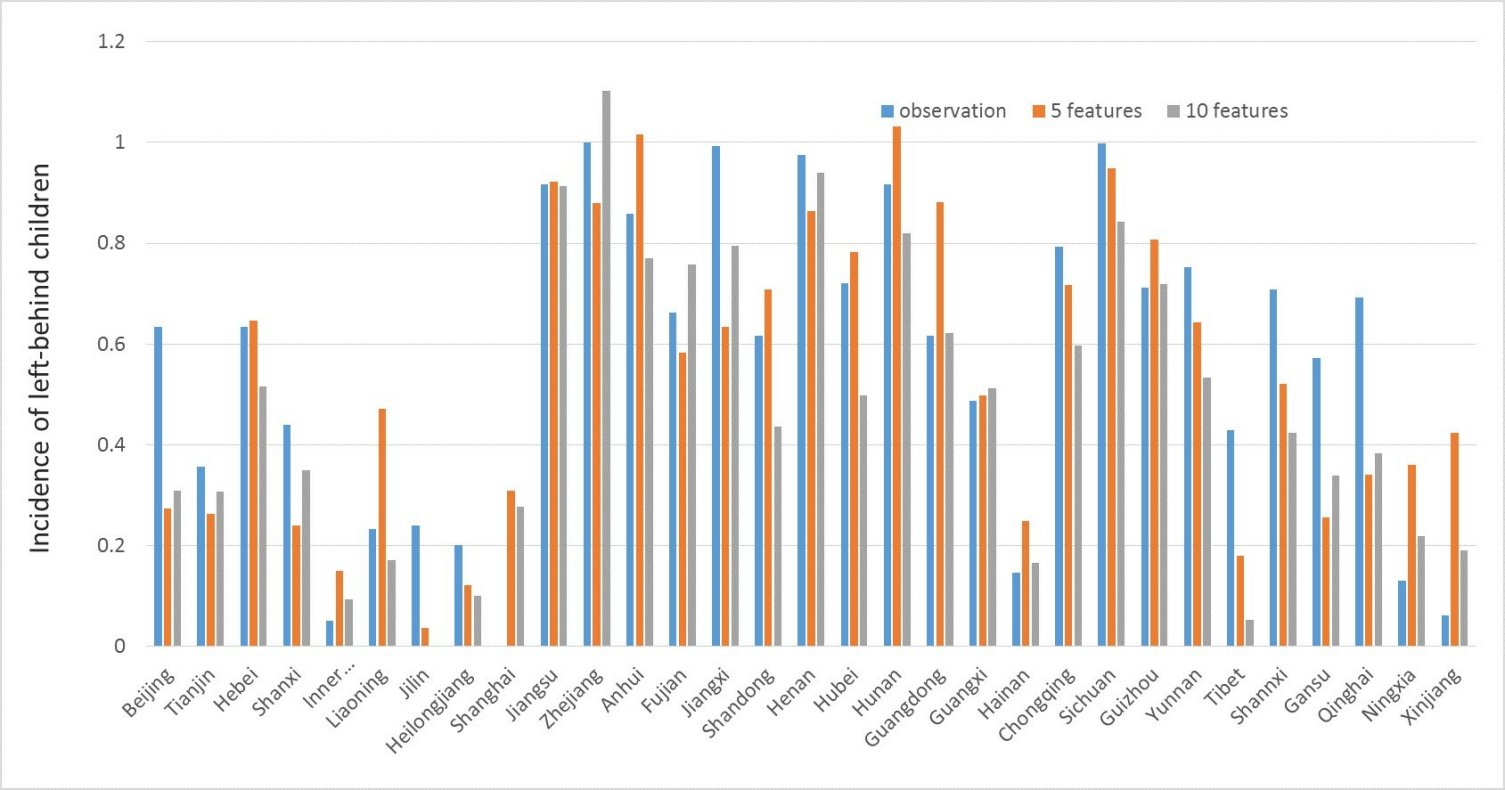
Prediction results for SVR models by filter method.

*Prediction of SVR models*. For Filter Approach, the top 5 and 10 features filtered by *r*^2^ measure are used. We choose models with higher *r*^2^ values for more accurate results then visualize both the predictive values by these models and the observations for each province in a chart. The prediction results are presented in Fig 5 and are compared against observation data.

When modelling, all the features and the targets have been normalized, So the values we predict are normalized numbers of incidence of left-behind children. Although these exact values are scaleless, they can still show relative relation between different provinces. For values less than 0 or greater than 1, it is reasonable since they are not the absolute values of the incidence of left-behind children.

Fig 5 shows the predictive values obtained by 5 and 10 features’ SVR models with the observed values. Generally, both models replicate the distribution of incidences. It is apparent that incidences are relatively high in central and eastern China, while relatively low in the northeast, northern and western China. However, for a certain province such as Xinjiang in northwest China, the result is unsatisfactory.

#### 2.3.2 Wrapper Approach and modelling

To predict the incidence of left-behind children on the province level, the Wrapper Approach is only used for province-level Data. The Wrapper Approach algorithm has two parts–the Search Part and the Evaluation Part. We first search for a subset of features, then evaluate it by some models and measures. By the way, we don’t need methods like in Filter Approach to select features here. For the search part, due to the huge space of feature subsets, some search strategies like Genetic Algorithms (GA) [30–33] and the K-means clustering method should be included. For evaluation, the prediction performance of models—*r*^2^ of the predictive values and the actual targets are used. The purpose of the evaluation here is to give suggestions on which features should be selected. In order to find high-score subsets of 5 or 10 features, some details of the algorithm need to be determined when the Wrapper Approach is implemented.

Search part

1 Genetic Algorithm Design

**Fig 6:**
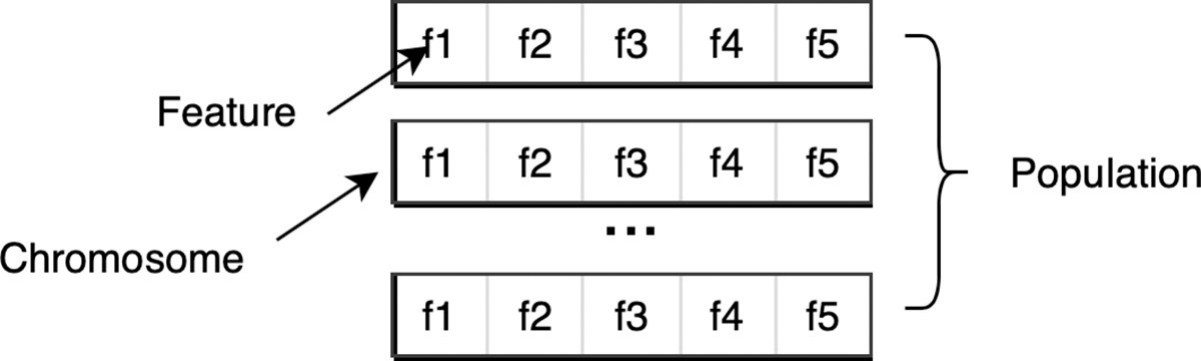
Features, chromosomes and population.

The basic idea of the genetic algorithm [34] is to construct a set of chromosomes (feature subset in this case), updated for each generation. Features that perform better have a larger possibility to be kept into the next generation. Conversely, poorly-performing features are less likely to be inherited. The relationship between features, chromosomes, and population is shown in Fig 6. The pseudocode for the algorithm is described in Algorithm 1.

**Algorithm 1**. **Search for feature subset by genetic algorithm.**

1: Generate the population of feature subsets *F* randomly

2: repeat

3:     **for all** chromosome *c* in *F* **do**

4:         Build regression model with *c*

5:         Evaluate fitness function *f(c)*

6:         **if** *f(c)* > *threshold* **then**

7:             Return the chromosome *c*

8:     Select parents *p* from *F* according to *f(c)*

9:     *k* ← Crossover(*p*)

10:     *k* ← Mutation(*k*)

11:     *F* ← *k*

12: **until** Termination conditions reached

The details are listed below:

- The chromosome here consists of 5 or 10 feature codes, which range from 0 to 110 for each. The population is set to be 5.
- Generate 25 integers range from 0 to 110 randomly for 5 chromosomes. These chromosomes are the first generation.
- The fitness function used in the procedure is the performance of the model built with the features in the chromosome. The performance of the model is the *r*^*2*^ value between the prediction and the true values.
- For each successive generation, the parents are selected by a proportion measured by the fitness function. Those with higher values of fitness function are more likely to be selected. For example, if there are three chromosomes scoring 0.2, 0.3, and 0.5 for the fitness function, respectively, the possibilities for these chromosomes being selected are 20%, 30%, and 50% respectively. 3 parents are selected for producing 5 new feature subsets.
- For the crossover, a one-point cross-over is used. The first three and last two features are swapped with each other for different parents. A mutation rate of 0.3 is applied so that there is a 30% chance that one of the successive generations would mutate one of its features randomly.
- The algorithm terminates when one of the following conditions has been reached. 1) The evaluation of some chromosome could reach 0.8, which means the performance is good enough. 2) The number of generations reaches a fixed bound. This condition is set to avoid an endless loop.

2 Search Based on K-means Clustering

**Fig 7:**
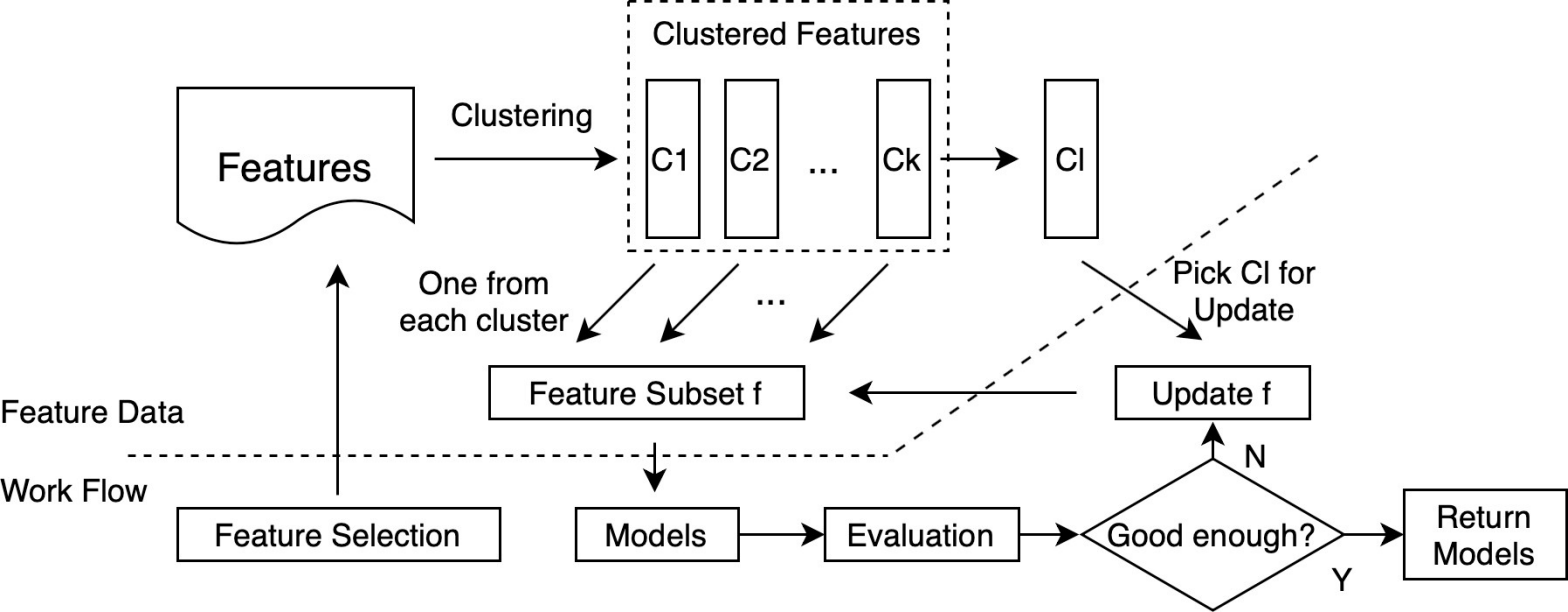
Clustering-based search.

The main idea of K-means Clustering [35] is to cluster features and select one from each cluster to represent the others. The number of clusters depends on the size of the feature subset. If the feature subset consists of 5 features, the number of clusters should be greater than or equal to 5. The workflow of the Clustering-based Search is shown in Fig 7. The feature subset is initialized by choosing one feature from each cluster. After that is an iteration. For each loop, the feature subset is simply updated by one feature randomly. If we assume the number of clusters equals the size of the feature subset, the algorithm is stated in Algorithm 2.

**Algorithm 2**. **K-means-clustering-based feature subset search algorithm.**

1: Initialize a feature subset list f

2: Cluster the original features into k clusters C = {ci}, i = 1,…, k

3: **for all** ci in C **do**

4:     Sample an integer m from 0 to len(ci) − 1

5:     Append the feature ci[m] into list f

6: **repeat**

7:     temp ← f

8:     Sample an integer l from 0 to len(f) − 1 to pick a feature from f

9:     Sample another integer m from 0 to len(cl) − 1

10:     f[l] ← cl[m]

11:     Build new regression model with new f

12:     Evaluate the new model

13:     if new model is worse then

14:         f ← temp

15:     Record f

16: **until** Termination conditions reached

The details are listed below:

- K-means clustering is used to cluster features. In each cluster, the features are re-indexed for the convenience.
- The old subset is kept. If the new value of the evaluation function is higher than the old value, then the subset of features is updated by the new one, otherwise, the update is not accepted. This process needs a repeat.
- Since iteration is needed, flip one of the features with certain possibilities at each iteration, which are proportional to the size of the cluster. For instance, if there are 3 clusters owning 2, 3, and 5 features, the possibility for each cluster to be selected is 0.2, 0.3, and 0.5, respectively.
- The algorithm terminates when one of the conditions has been reached. 1) The evaluation functions reach 0.8. 2) The number of iterations reaches a fixed number.

*Evaluation part*. As previous literature [27] mentioned that the Wrapper Approach consists of using the prediction performance of a given learning machine to evaluate the relative usefulness of subsets of variables. Since Wrapper Approach reconstructs the model for each iteration, a large number of models can be built during the process. The parameters of the models are set up in advance because It is impossible to optimize the parameters artificially for each model. The SVR models for both search strategies and numbers of features (5 or 10) are set up by the same parameters listed in Table 9. The parameters of NNR models for different search approaches and feature numbers are given as follows in Table 10.

**Table 9:** Parameters for SVR model by Wrapper Approach.

Trade-off Constant C	Kernal Function
100	RBF

**Table 10:** Parameters for NNR model by Wrapper Approach.

Number of Hidden Neurons		Loss Function	Learning Algorithm	Learning Rate	Coefficient of L2 Regularization	Activation Function
5 Inputs	10 Inputs	MSE	SGD	0.01	0.01	Relu, Tanh
5	8					

The experiment is repeated 10 times for both kinds of models. For SVR models, each time, the iteration limit is set as 300 and one of the top 30% validation value results are randomly picked. For NNR models, the experimental procedures are similar to SVR, except for the number of iterations set to 30, because the training and validation of neural network models take a much longer time. Five times average performance is the final evaluation result to compare because of five-cross validation. The average performances of SVR and NNR models built by Wrapper Approach are listed below in Tables 11 and 12.

**Table 11:** Average performances for SVR models built by Wrapper Approach.

Feature selection methods		Performance	
Number of features	Search method	*r*^2^	MSE
**5 features**	GA	0.3733	0.0584
	K-means clustering	0.5961	0.0371
**10 features**	GA	0.4261	0.0532
	K-means clustering	0.5352	0.0432

**Table 12:** Average performances for NNR models built by Wrapper Approach.

Feature selection methods		Performance	
Number of features	Search method	*r*^2^	MSE
**5 features**	GA	0.3840	0.0574
	K-means clustering	0.4546	0.0510
**10 features**	GA	0.3928	0.0563
	K-means clustering	0.5122	0.0451

1 Support Vector Regression

According to Table 11, it is obvious that the K-means clustering based search can give a higher value of *r*^2^ than the genetic algorithm. Besides, it is still possible to use fewer features to build better SVR models.

2 Neural Network Regression

According to Tables 11 and 12, the search method based on clustering shows better performance than the genetic algorithm, and the SVR models built by differently selected features appear more stable for test performance. Therefore, for the Wrapper Approach, it is more suitable to use the K-means clustering-based method to build SVR models.

*Prediction of SVR models*. After the Search and Evaluation Part, we have got models with good performance and the corresponding feature subsets. We simply choose one of the high scoring feature subsets as an example to represent the features obtained by this approach. The selected 5 and 10 features are listed in Tables 13 and 14.

**Table 13:** 5 features by Wrapper Approach.

	Feature
**1**	residential real estate development investment
**2**	number of motorized thresher
**3**	construction employments in urban units
**4**	national special entitled groups with regular subsidy
**5**	student-teacher ratio of primary school

**Table 14:** 10 features by Wrapper Approach.

	Feature
**1**	construction area of residential house
**2**	number of domestic design patent application examined
**3**	mainland residents registration of marriage
**4**	hospital bed using-days
**5**	number of special education students
**6**	number of performing arts groups
**7**	fresh vegetables consumer price index
**8**	national special entitled groups with regular subsidy
**9**	Average wage of employees in urban units
**10**	highway passenger transportation

As shown in Tables 13 and 14, these two feature subsets are representations of feature combinations obtained from our Wrapper Approach and Modelling Section. And they are assumed to help build good SVR models. Two SVR models can be trained using these features and the parameters in Table 9. The performances of these two models are listed in Table 15. It is obvious that the performance of SVR models using feature subsets selected by the Wrapper Approach can be as good as the best model built by Filter Approach.

**Table 15:** Performance of two chosen SVR models.

	Performance	
Number of features	*r*^2^	MSE
**5 features**	0.7164	0.0264
**10 features**	0.7033	0.0276

**Fig 8:**
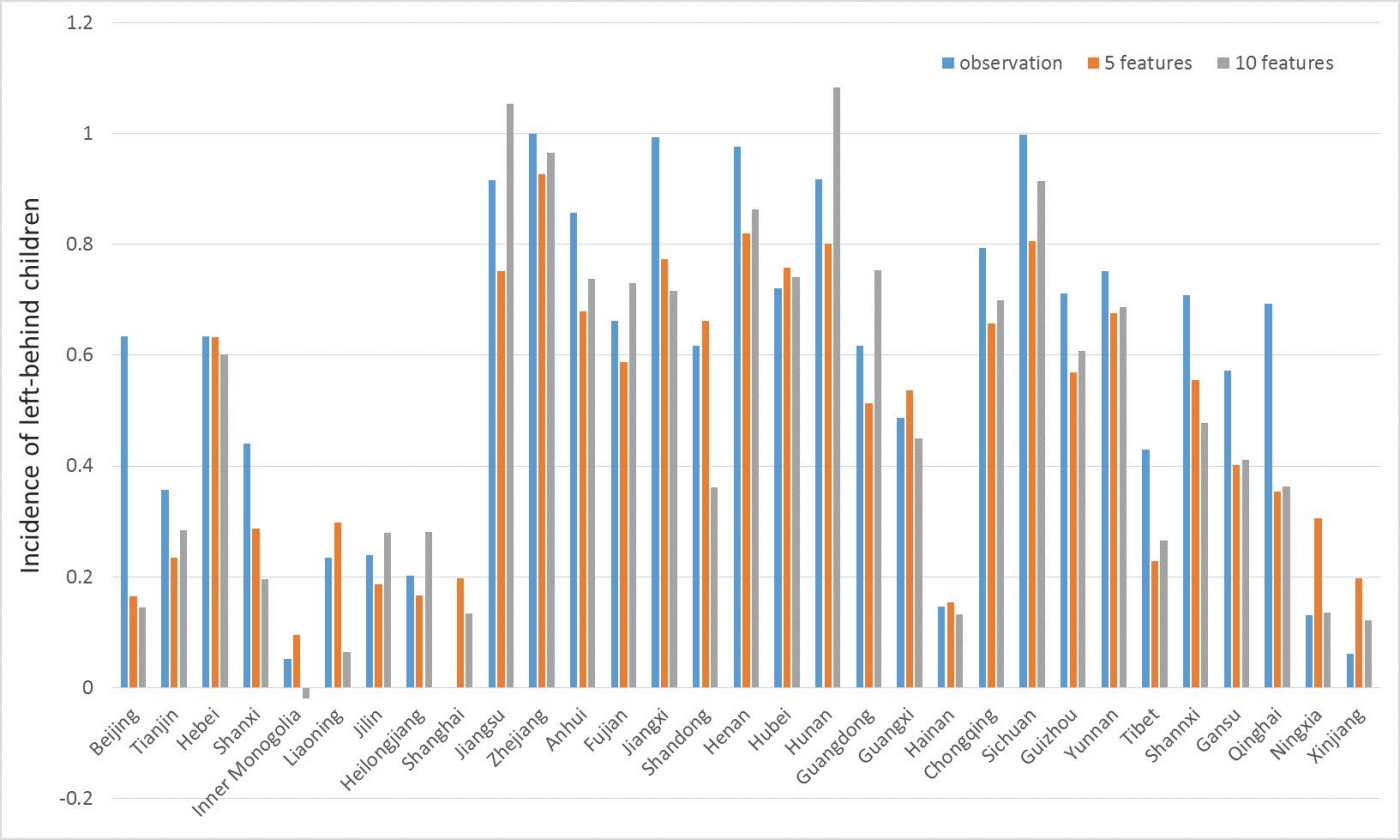
Prediction results for SVR models by Wrapper Method.

The two SVR models above are used for prediction. The results of the prediction are listed in Fig 8. As shown in Fig 8, for most provinces in the perspective of relative values, the prediction can give a correct result. Although the models may not be able to give accurate predictive values for each province, they could provide valuable information about the nationwide distribution of incidences and the relative values of incidences between provinces.

### 2.4 Assessment of the model

**Fig 9:**
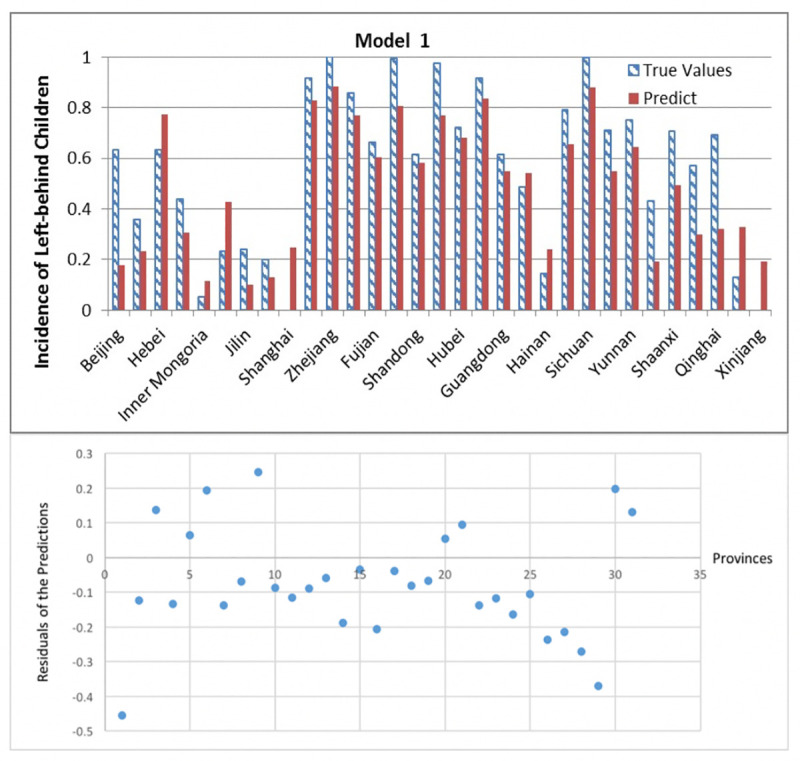
Prediction results and residuals of Model 1.

**Fig 10:**
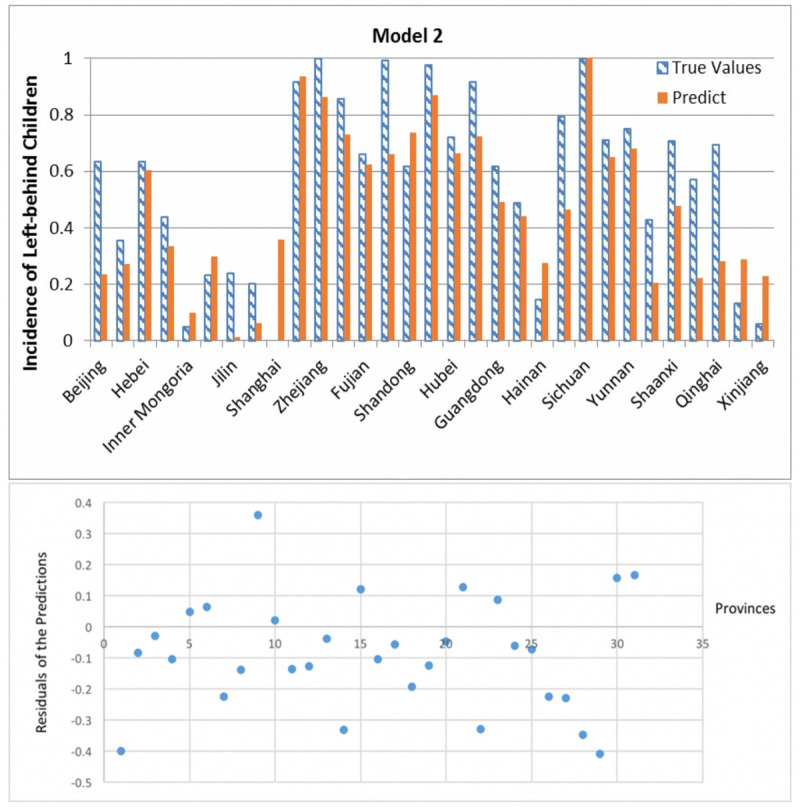
Prediction results and residuals of Model 2.

**Fig 11:**
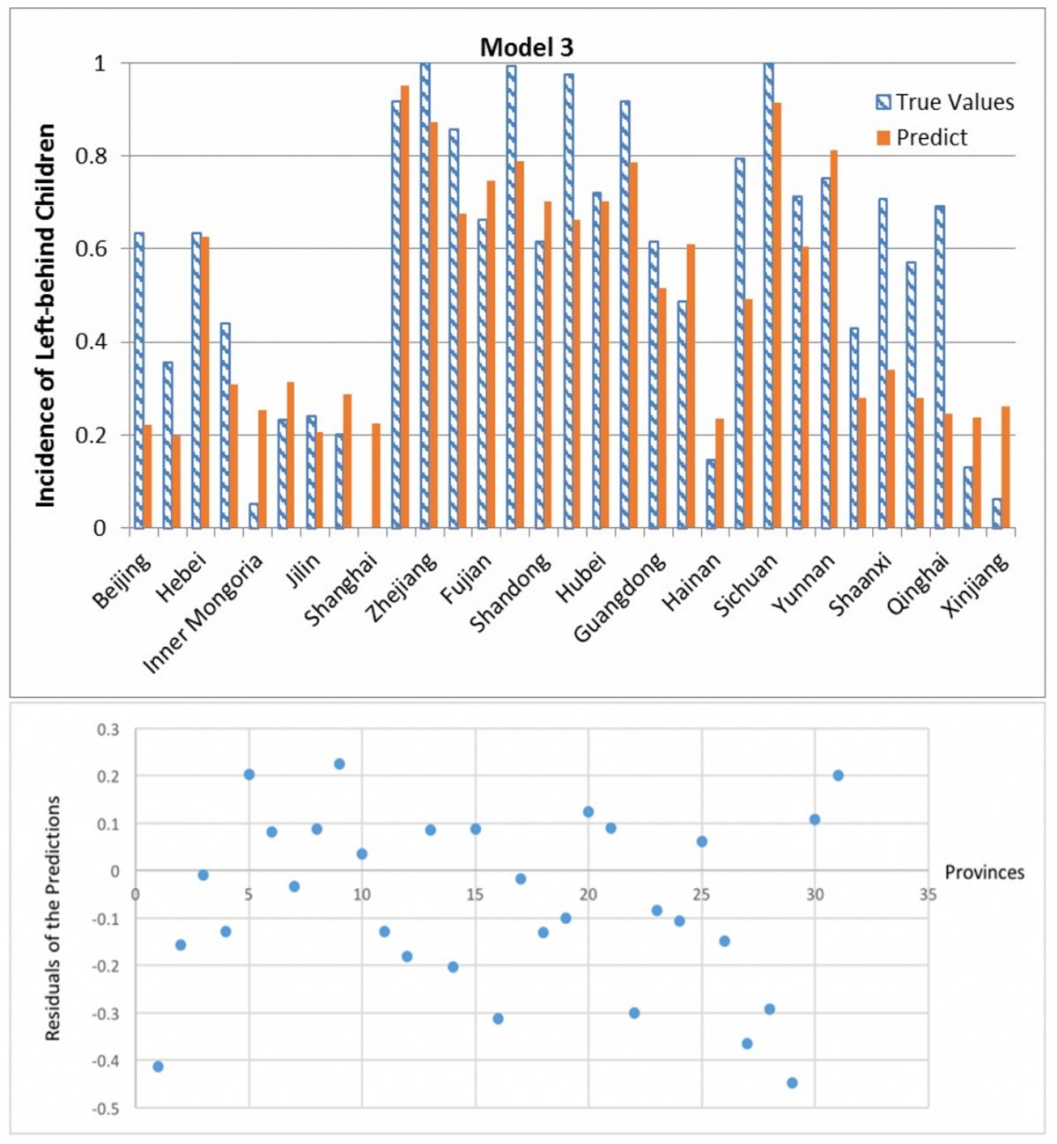
Prediction results and residuals of Model 3.

Another essential step in our workflow is to assess the models which work well. Based on the comparison results given above in the 2.3 Section, we construct three specific SVR models using three feature subsets got from the k-means clustering-based method respectively, because we conclude that such models can perform well in our case study. The features of these three models can be found in S1 Appendix: Assessment models’ features. Besides studying the average performance of models, these three specific models are analyzed through the residual plots here in Figs 9–11. By analyzing their prediction performance, we can know exactly how well the models perform.

In nearly all traditional regression models have residual greater than 0.4. According to the residual Figs 9–11, the three models’ absolute values of residual are all less than 0.4, where residual means the difference between real data and prediction. And it shows the residual is less spread out between 10–25 provinces in all three models. What is more important is that these results are difficult to achieve in traditional regression methods.

### 2.5 Voting for key fields

The aim of the VFK procedure, which is the final step of our workflow, is to select the key fields which are more related to the target variable. The voting process is done according to the results of the Wrapper Approach. In the Wrapper Approach, the experimental results show that many feature subsets perform well. Therefore, implementing some statistical method is proposed to explore whether some fields matters more.

**Fig 12:**
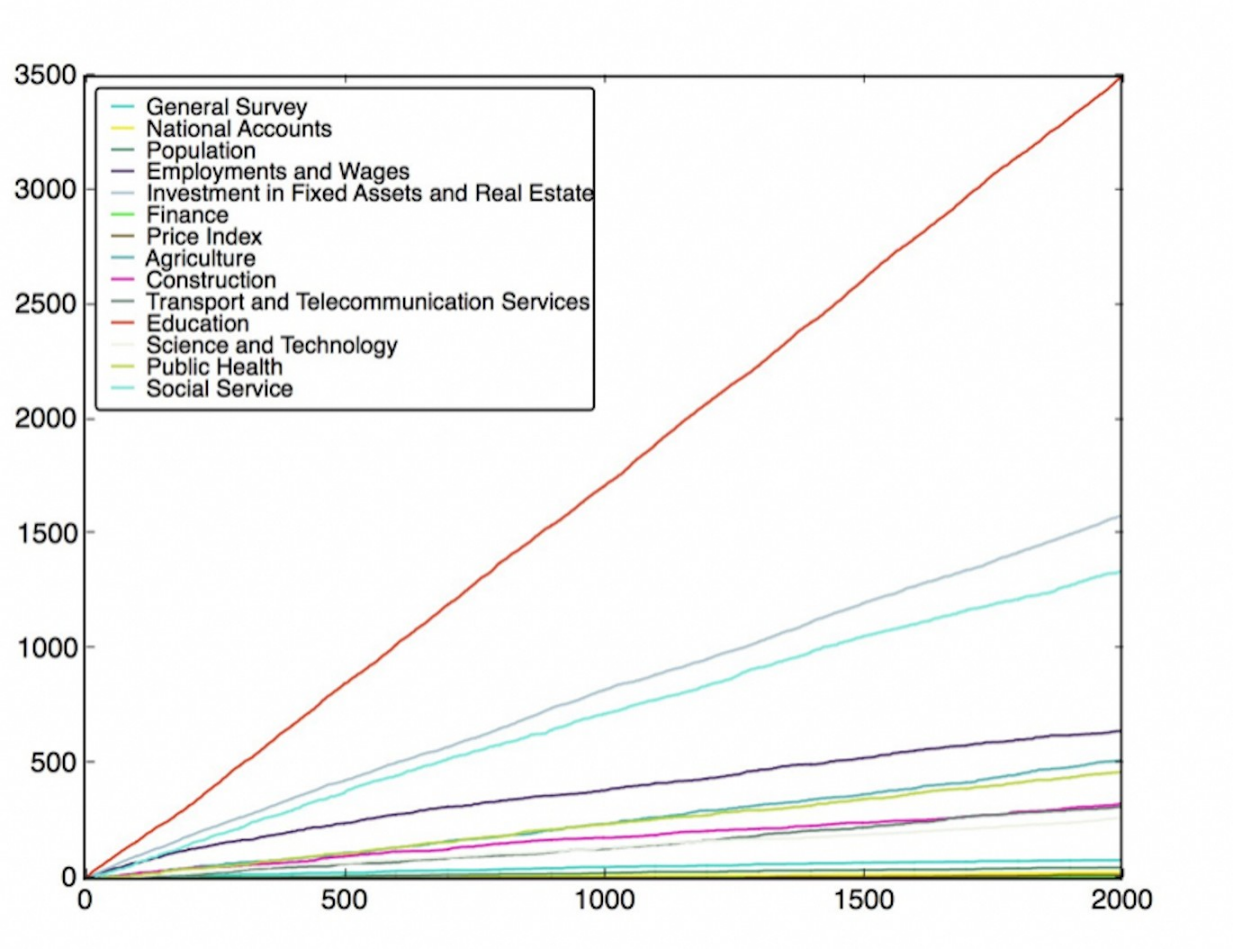
SVR with 5 features and clustering method.

**Fig 13:**
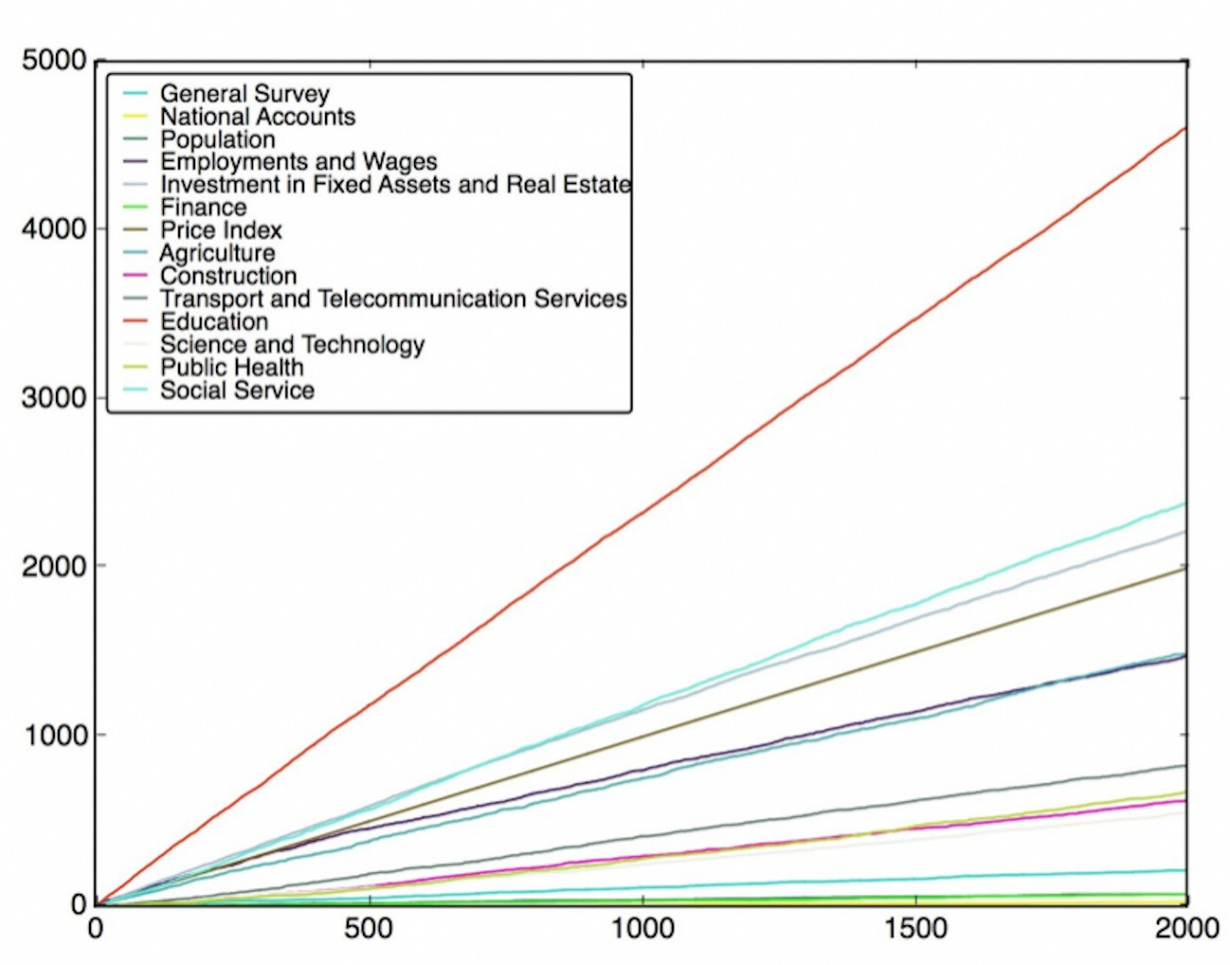
SVR with 10 features and clustering method.

**Fig 14:**
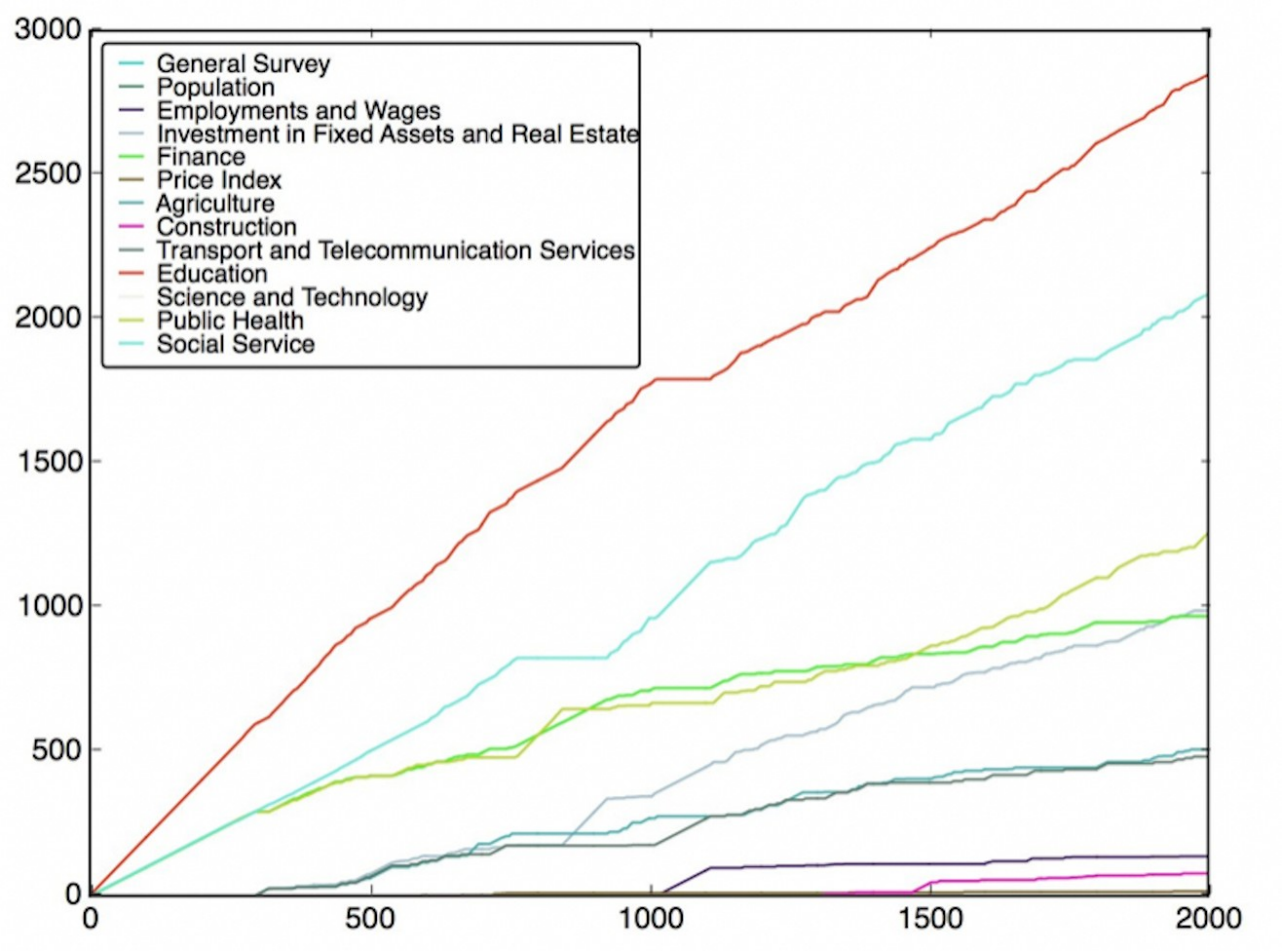
SVR with 5 features and genetic algorithm.

**Fig 15:**
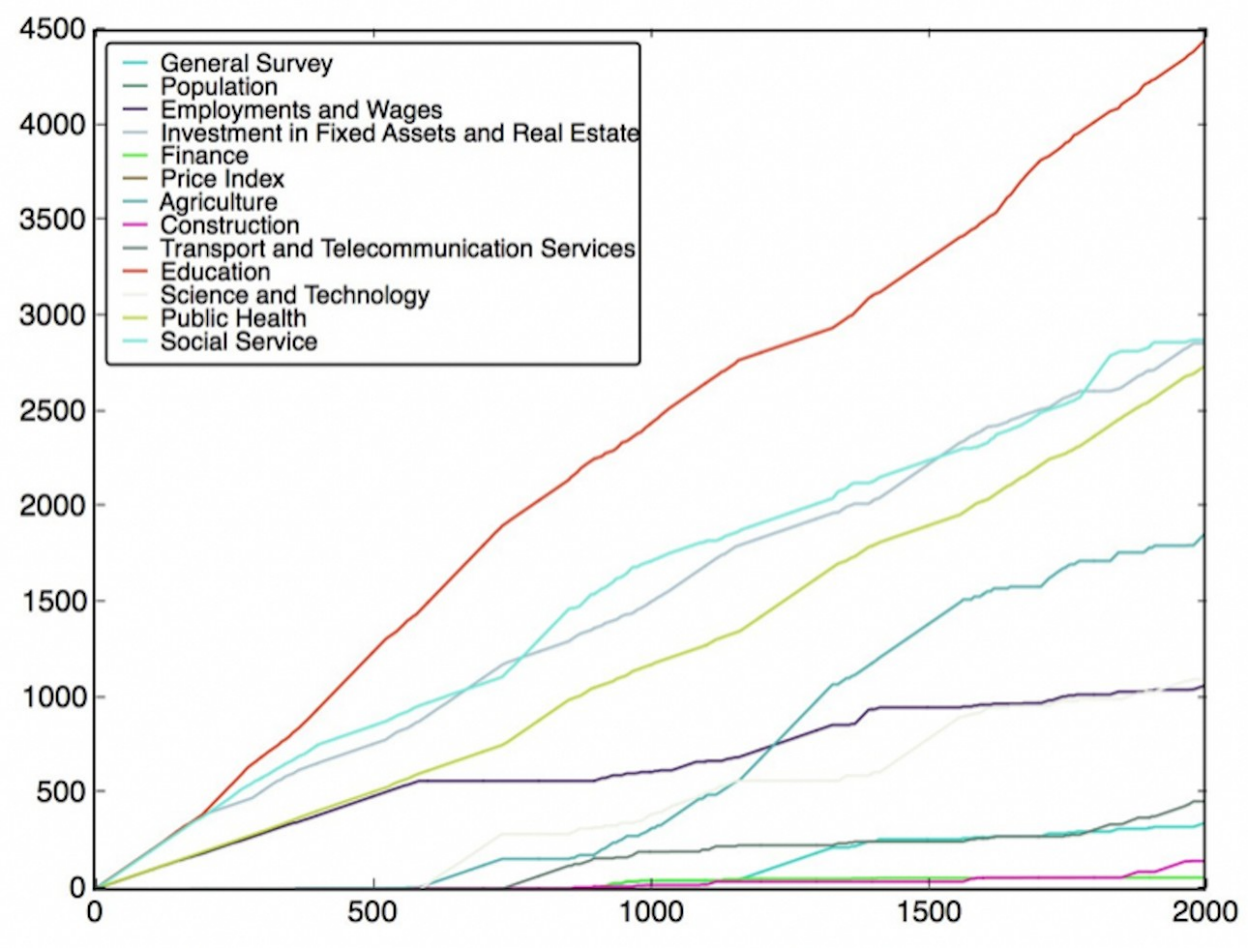
SVR with 10 features and genetic algorithm.

**Fig 16:**
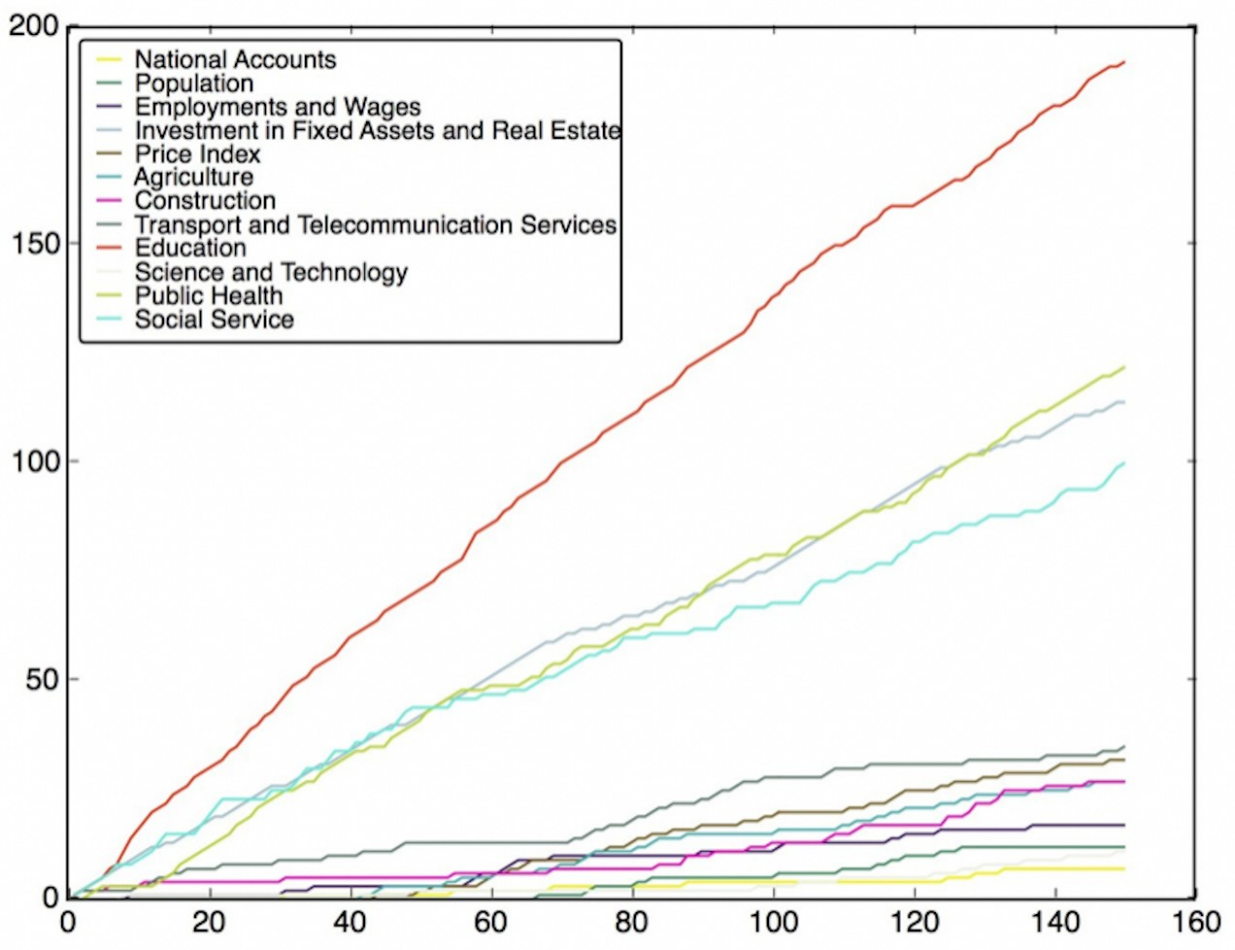
NNR with 5 features and clustering method.

**Fig 17:**
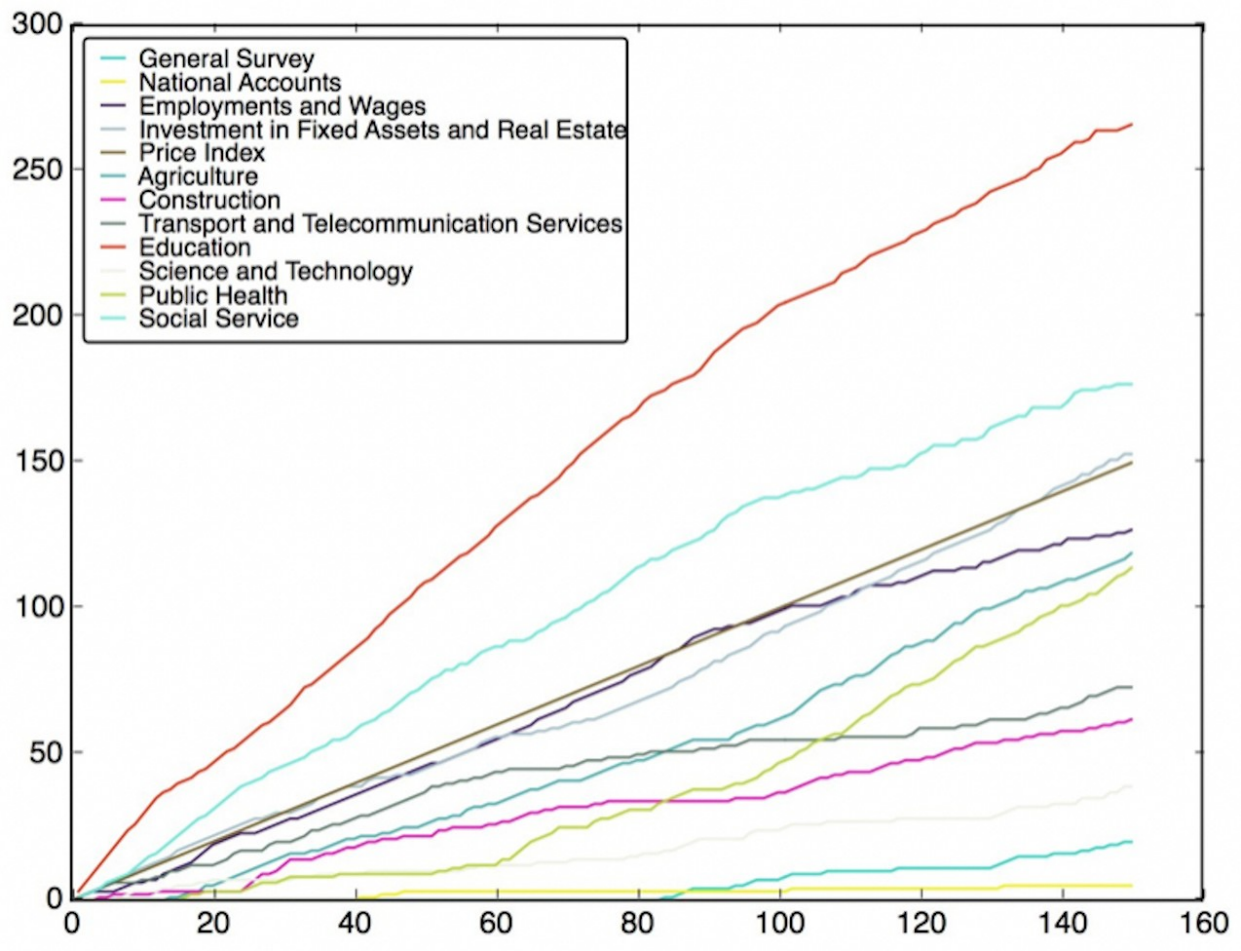
NNR with 10 features and clustering method.

**Fig 18:**
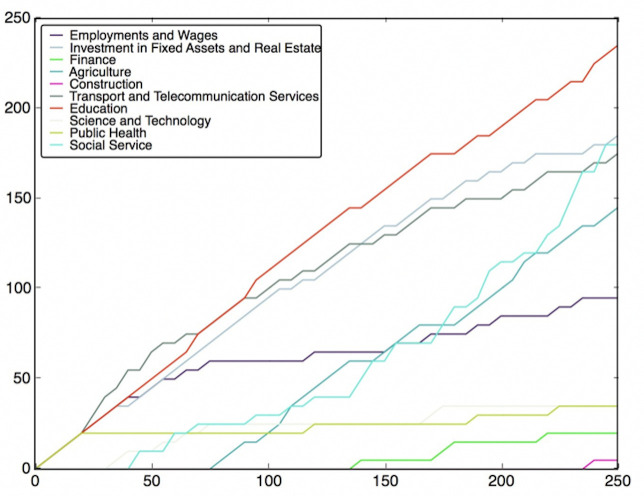
NNR with 5 features and genetic algorithm.

**Fig 19:**
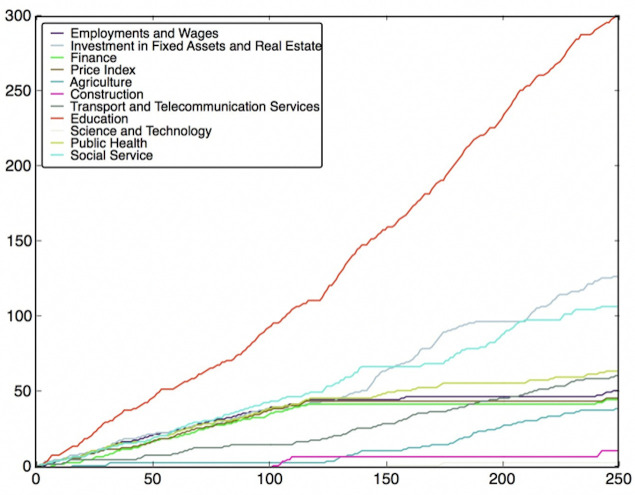
NNR with 10 features and genetic algorithm.

To do this, the features are classified into different classes (fields), which have been listed in Table B in S1 Appendix. Then, we need to use the feature subsets got from the Wrapper Approach. For every round of voting, a feature subset is used. For each feature subset, the features vote for their own classes according to their model performance. After voting with all feature subsets, the charts of the fields’ score versus voting rounds can be plotted, which are shown in Figs 12–19. If some classes’ scores increase faster than others and the trend of the increase is maintained, these fields may be considered as the most relevant fields that impact the target variable—the incidence of left-behind children. The plots of different models are shown below. For each group of pictures, the first one is of the model with 5 features and the second one is of the model with 10 features.

As shown in the Figs 12–19, the most attractive result is the ‘Education’ field, which stands out in almost all cases as the number of selected models accumulates. We can draw the conclusion that education is the most significant field that impacts the incidence of left-behind children at the province-level. Besides, Social Service, Investment in Fixed Assets and Real Estate, Public Health are also significant fields. Comparing different methods of feature subset search, the clustering-based search method shows a relatively stable proportion for each field as the total count increases, which means that the key field can be determined with fewer attempts. Also, the result of SVR model is more stable and reliable than NNR model.

## 3 Result

### 3.1 Scenario simulation

From the perspective of reviewing the performance of our workflow, we conduct a scenario simulation to investigate the impact mechanism of education, because according to the experimental results education can be considered as the most significant field relevant to the incidence of left-behind children at the province-level.

**Fig 20:**
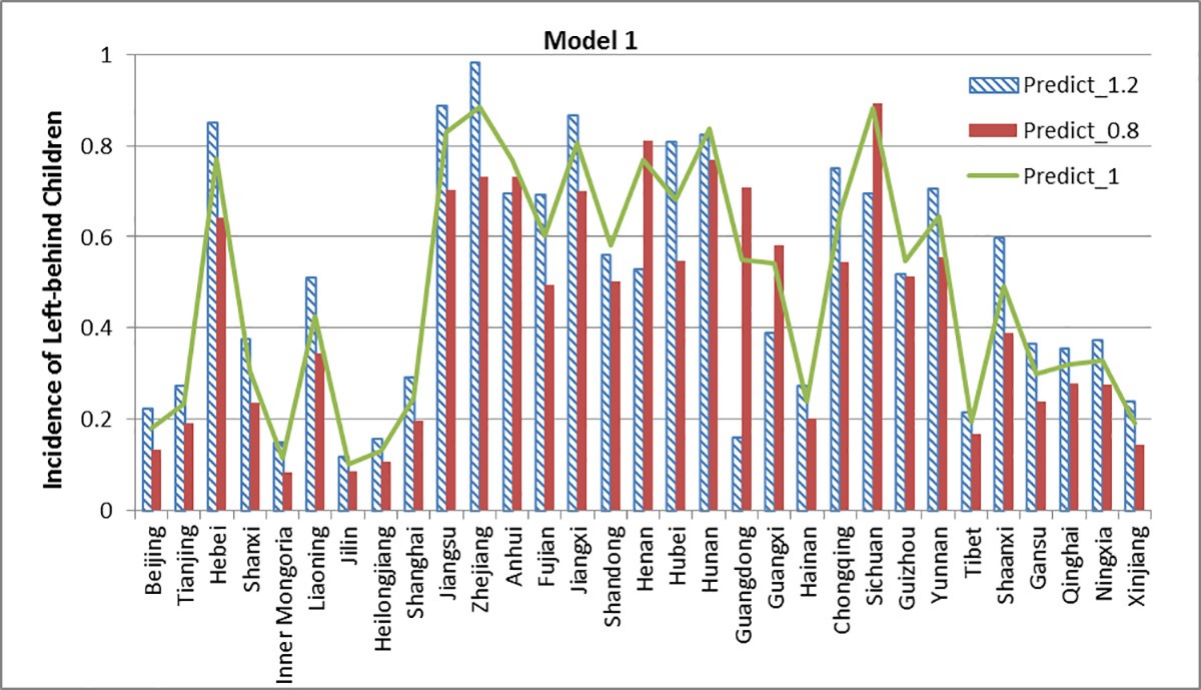
Simulation results of Model 1.

**Fig 21:**
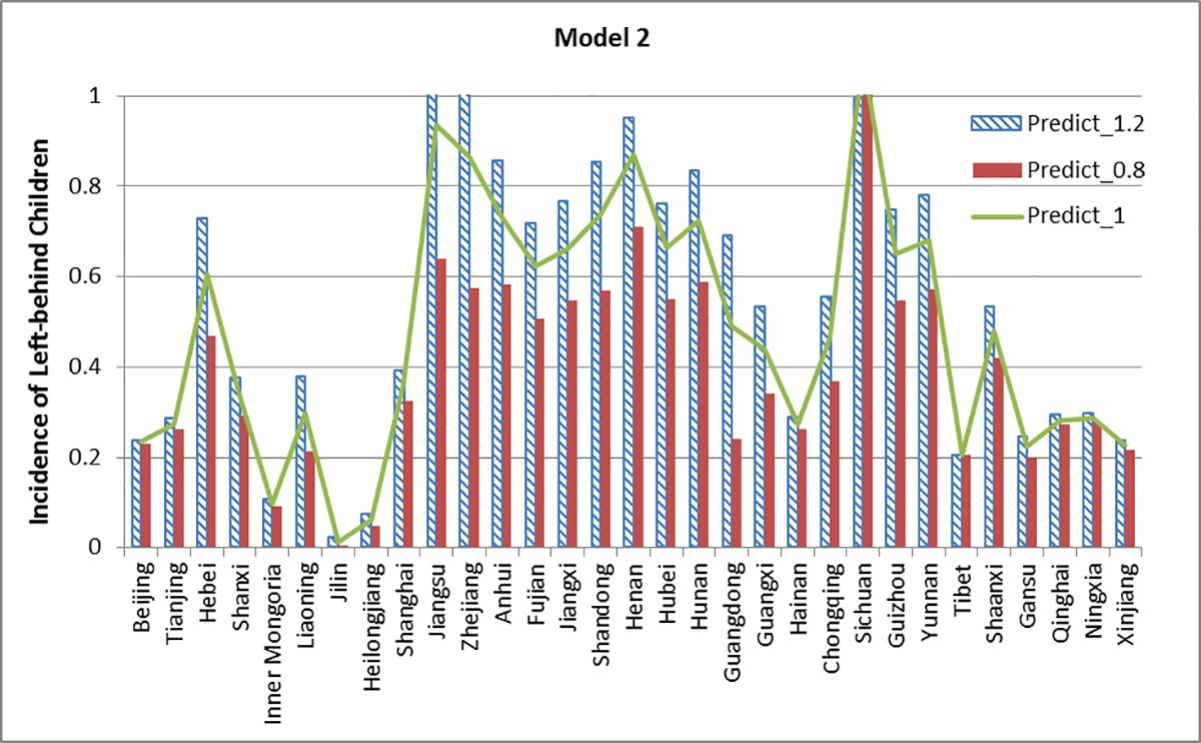
Simulation results of Model 2.

**Fig 22:**
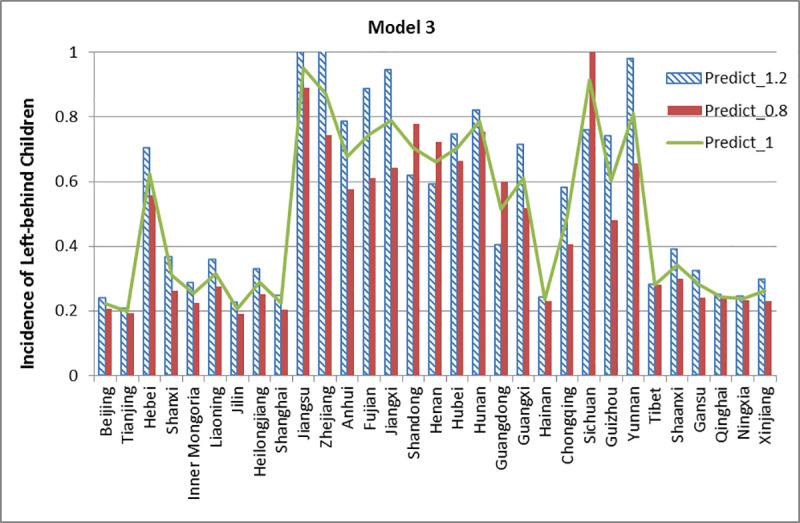
Simulation results of Model 3.

The three models from the section “Assessment of the Model” are used to simulate and compare the incidence affected by different education levels. Model 1 and 2 contain three educational features, while model 3 contains two educational features. We expand the educational features to 1.2 times their original values and shrink them down to 0.8 times, other features are kept invariant. Then we compare the results of prediction. The results of the simulation are shown below in Figs 20–22.

Fig 20 shows that the incidence of left-behind children of 23 provinces has improved when the relative importance of educational features is increased by a factor of 1.2. The incidence of left-behind children of 27 provinces reduces to some extent under 0.8 times of educational features. It is interesting to note that Henan, Guangdong, Guangxi, and Sichuan have the opposite results, which means that the incidence of left-behind children at these four provinces decreased under high-level educational features and vice versa.

From Fig 21, we find that the incidence of left-behind children of 29 provinces has improved when an increased weighting is given to educational features. The incidence of left-behind children has decreased when these features are under-weighted, except in Tibet. As the true value of Sichuan is close to 1, the predictions under different situations are also close to 1. Interestingly, the predictive left-behind incidence of Tibet maintains unchanged (0.2048) for different situations, lower than the true value (0.4297).

In model 3, it can be seen that the incidence of left-behind children of 26 provinces has increased with a higher weighting of educational features. By contrast, the incidence of left-behind children reduces with a lower weighting. Similar to Model 2, the predictive left-behind incidence of Tibet maintains unchanged (0. 2797) for different weightings, lower than the true value (0.4297). It is interesting that Guangdong and Sichuan have the opposite results, which means the incidence of left-behind children of these four provinces decreased under high-level educational features and vice versa.

To sum up, there is a positive correlation between education and left-behind children’s incidence for most provinces. However, the incidence of Henan, Guangdong, and Sichuan negatively correlated with education. Also, the change of educational features has no appreciable effect on Tibet’s incidence. Therefore, it is reasonable to presume that increasing educational input will attract more children from migrant families to come to receive education in the census register seat. We suspect that as Tibet is sparsely inhabited, economically backward that lags behind its educational standards so that the changing scope of educational features is too small to influence the Tibet’s left-behind children incidence. Therefore, the predictive left-behind incidence of Tibet maintains unchanged for different situations in model 2 and model 3. It is possible that there exist some significant features that influence the incidence of left-behind children in Tibet that have not been discovered yet. Additionally, the factors contributing to a negative correlation between the left-behind children’s incidence and educational features need to be further investigated.

### 3.2 Conclusion of case study

The quantitative analysis of left-behind children is considered to be challenging but rewarding work. After applying a series of data mining approaches to the migration data, the conclusions we obtained can be summarized as follows.

First, we design and implement feature selection approaches, which are applied to Province-level Data. For the Filter Approach, different measures including Pearson’s correlation coefficient, the coefficient of determination, and maximal information coefficient, could have high-performance models in different cases. There is no dominant measure of the experiments we conducted. For the Wrapper Approach, however, the average performance of the k-means clustering-based method is always higher than that with the genetic algorithm method. Therefore, the k-means clustering-based method is recommended firstly when trying the Wrapper Approach.

Second, as for the models built for prediction [36], SVR models perform better than models no matter which approaches are chosen for feature selection. Besides, the time of training SVR models is much shorter than training NNR models for the data set. Therefore, for this problem, SVR is more suitable for both modelling and feature selection purposes.

Third, in section 2.3, four SVR models built with four subsets of features, which were selected by the Filter and Wrapper Approach respectively (with 5 and 10 features for each approach), have been trained to predict the target. The performance, *r*^2^ values, of the models are 0.5788, 0.6715 for the Filter Approach, and 0.7164, 0.7033 for Wrapper Approach. Therefore, we randomly selected three specific models from SVR models with Wrapper Approach. The results of the prediction show these models are relatively accurate. We also find a positive correlation between education and left-behind children incidence for most provinces in China through scenario simulation.

Last but not least, we find that the most significant field related to the left-behind children’s problem is education. According to the results of the analysis on the migration data, for a family, the education level of parents and the province where migrants come from (place of huji—registered permanent residence) are the top two relevant features of whether left-behind children might occur. This may be a reason that education-related policies should have high priority over others to deal with left-behind children’s problems in different provinces. Besides, Social Service, Investment in Fixed Assets and Real Estate, Public Health are also significant avenues for future policy.

## 4 Discussion

To construct a general methodological workflow, we need to provide solutions to general problems so that other researchers can follow them. Several discussions about technical details in the workflow are below.

First of all, the number of features selected should be as small as possible so that a relatively easier explanation can be provided and a smaller workload of data collection be required for prediction. However, if more complex models must be used, a higher dimension combination of features might be required. To find an optimal number of features we may need to test with specific models. If the model performance does not improve significantly when increasing the number of features, this should be considered as an approximate upper bound.

Secondly, dimensionality reduction of feature space may be needed when designing the algorithm. In our algorithm design, methods such as Principle Component Analysis (PCA) does not be used, as we believe that PCA simply uses few components to represent the original data, which is inconsistent with our purpose of feature selection. And we believe that all the selected features should be themselves rather than the linear or other combination of original features. However, some previous research using principal components as input variables for prediction [14] makes a comparison of different regression models. While this methodology may be possible to implement, but we leave that to future work.

Our third question is the number of clusters. For clustering-based search, the number of clusters could be greater than the expected size of the feature subset. If 5 features are needed, the original features can be clustered into 5 groups. More clusters can reduce the error caused by the clustering algorithm, but this will increase the number of features selected. Therefore, we can choose a relatively large number of clusters first. Then, we analyze the correlation of the cluster center with the target and select part of the most relevant clusters. Finally, we use the selected clusters to complete the following steps described above. This could be an efficient method for small subset selection as well and might do in future work.

The fourth question is about identical features that may appear in a feature subset. Consider the possibility that two features are repeated when selecting m features from n features. The possible combination number is n^m^ in total, while there are mn^m-1^ kinds of possibility if and only if one feature repeatedly appears once. Thus, the approximate probability of repetition is: *P* = $\frac{mn^{m-1}}{n^{m}}=\frac{m}{n}$. In this case, n = 100, and m is chosen to be 5 and 10, so P is less than 10%. Therefore, we can deal with it simply by abandoning the solution, which means about 10% extra time is required. By comparing with the workload of a selection algorithm without repetition, we find this to be acceptable. Besides, the feature subset generated by clustering based search avoids this issue.

The last question is about the cost of increasing predictive accuracy, which may limit the explanatory power and interpretability—the key issues for social scientists. Sometimes, it may be difficult to understand a neural network and link it with previous social science research results. For example, research showed that the neural network approach is parsimonious, produces better classification, handles complex underlying relationships better, and is stronger at interpolation. However, NNR models may lack interpretability at the model structure and weights [37]. Despite the above challenge, we believe such methods have great applicability to social science and will show better predictive ability than traditional methods as previous literature proved. Moreover, the research found that neural network is particularly suitable for learning nonlinear functional relationships or multiple output and input relationships which are not known or cannot be specified [15].

## 5 Conclusion

### About general workflow

In this paper, we construct a relatively general methodological workflow that can be used to conduct research on social issues. In our workflow, we show the performance of both feature selection methods—Filter and Wrapper Approach and models with NNR and SVR prediction results. Besides, we check three models to see how well the models are through the residual plots. Further, the VKF method provides us an important ranking of different variables. In section 3.1, the simulation provides an understanding of the prediction scenario of policy intervention.

The methods we use in our workflow are different from the traditional methods usually implemented in social science such as multiple linear regression, instead, we transfer methods from machine learning techniques. Not only because the methods can better fit the non-linearity of the data set, but also these methods can provide new insights not restricted by the former knowledge and theories. This can happen because the process of feature selection is not based on prior knowledge or literature review, but based on the data characteristics instead. From this perspective, without the limitation of prior knowledge, our general methodology can conduct research in unfamiliar fields as long as we have available data sets. However, we do not refuse any prior knowledge, as experience or theories can help to explain the result.

Also, we make possible predictions based on selected features and models that can provide a future scenario for what might happen. What we want to emphasize here is that traditional social science research focuses on the effectiveness of parameters instead of prediction, however, prediction accuracy [38] can also provide a clue to understanding social issues. In addition, we provide a ranking of the features to provide clues of which field is relatively more important to the target variables. Although these are of statistical significance, they can also be used in reference to decision making and policy formulation. With the explanation of theory and knowledge of the related field, some policy interventions can be provided.

### About case study

As this paper focus on the left-behind children’s issue, full details about the case study can be found in the S1 Appendix. Regarding left-behind children’s problems, the methodology we use is coherently suited to catch the non-linearity behind, and we briefly summarize our contributions as follows.

First, compared with former research on left-behind children [39–41], there is no research about the incidence rate at the province-level in China. However, it is clear that plenty of further research can be conducted based on the incidence rate, because it is a fundamental and macro-level factor that explains the left-behind children’s problem.

Second, we create a linkage between two different data sets, namely Migrants Population Dynamic Monitoring Survey Data [42] provided by the National Health Commission, and the Province-level Data from the National Bureau of Statistics of the People’s Republic of China. We have not found any prior research that brings these two data sets together, rather most studies use the Population Census Data from China [40], which is an open-source data set instead.

Third, based on the new methods and data sets’ linkage created, we can make some relatively accurate predictions on the incidence rate of left-behind children. However, we cannot guarantee the accuracy of each province, but we may provide a relative trend of individual province and the distribution of all provinces. Moreover, as we focus on automated workflow design, we do not focus on the accuracy comparison of machine learning techniques and traditional regression methods.

Forth, with the VKF analysis, we find the ranking of significant fields that indicates their influence on the incidence rate of left-behind children, such as Education plays the most important role in different models. Besides, other fields such as Social Service, Investment in Fixed Assets and Real Estate, and Public Health are also important but not that important compared with Education.

Fifth, the scenario simulation shown in Figs 20–22 can provide a possible prediction status on what will occur if the dominant factors are changed, which is important for policy intervention. For example, in the models we selected, the scenario simulation proves that when the proportion of education-related features increased, the incidence of left-behind children is increasing in most provinces and decreasing in a few provinces. Under different circumstances, the government should provide different interventions in order to achieve the goal. However, there is still some complex mechanism underlying why the change of education has diverged influence on incidence in different provinces that will be left to further research, which may need to combine some push and pull factors [43, 44] and regional differences.

## 6 Future work

As for future work, we can improve from the following perspectives.

From the perspective of methods, the details of the design and implementation of wrapper methods need further optimization. For example, within the genetic algorithm, the population, crossover, mutation strategies, and other parameters may be varied to improve the overall method. It is always hard to say a genetic algorithm search is implemented perfectly for every detail. Additionally, the k-means clustering-based search can be improved in several ways. One idea is that other clustering algorithms like expectation-maximization can be tried. Another is that we can choose the number of clusters higher than the number of features, and analyze the correlation of the center (or other ways to represent cluster)in each cluster so that we can determine how significant the cluster is to the targets and only use the relatively significant clusters to construct the feature set. Other options might be within the implementation of methods, such as using fewer features in SVR models, so that the model may be easier to recognize and explain with less mathematical computation and get better and clearer testing and prediction results.

From the perspective of data, if more data sets are available, such as multi-year data sets that link coherently, we can provide better training results based on machine learning methods. Additionally, if the city-level data are available, whose volume is bigger than the province-level’s, which may help interpret the incidence of left-behind children more precisely than Province-level Data.

Besides the above, as our methodology is a relatively general and automated one, we hope that it can be applied to explore other social issues. However, from the perspective of providing an explanation, we may try to incorporate the traditional methods such as Multiple Linear Regression or Logistics Regression so we can add more interpretability to our results. Then SVR and NNR models can be complementarily used with traditional methods.

## Supporting information

S1 Appendix (DOCX)
