# Supplementary material for: A data driven methodology for social science research with left-behind children as a case study
Source: PLoS One. 2020 Nov 20;15(11):e0242483. doi: 10.1371/journal.pone.0242483 (PMC7678991; doi:10.1371/journal.pone.0242483)
Supplement: S1 Appendix — (DOCX) [file pone.0242483.s001.docx]

# Case study: left-behind children in China

The definition of left-behind children is “0-17 years old children who have at least one parent living outside the household registration area due to his or her job location”. China has encountered mass labor force migration from rural to urban areas over the past several decades of urbanization and industrialization, and the number of left-behind children has dramatically increased. The latest data shows that left-behind children make up 35.6% of rural children. The incidence of left-behind children in inter-provincial outflows areas is as high as 43.8%, such as Anhui, Henan, and Sichuan.

The left-behind children issue is a serious problem for the lack of parental instruction and care during the formative period of these children. Recent research focuses on consequences caused by left-behind children from different perspectives with various methods. There are some assessment scales used to assess the mental health of this group, such as Children’s Depression Inventory-Short Form (CDI-S) [1], Pediatric Quality of Life Inventory [2]. Other quantitative methods were also applied, such as logistic regression [3-6], chi-square analysis, ANOVA and post-hoc analysis, cluster analysis, univariate and multivariate analysis [7,8], Kruskal–Wallis [9], propensity score matching (PSM) [10] and so on. These literatures focus on physical and mental health status, educational performance and the general well-being of left-behind children.

Our approach is different as we focus on influence features of the incidence rate of left-behind children, such as regional education level. We use data-driven methods from machine learning techniques to select and model all possible factors about left-behind children. To illustrate the problem, we create linkage between Migrants Population Dynamic Monitoring Survey Data and Province-level Data for the year 2013 coherently to understand the left-behind issue, which is the first time these two data sets have been used together to portray this issue. Although the incidence of left-behind children is an individual trade-off of cost and benefit analysis, the fundamental reason is the unbalanced development of the economy between urban and rural areas. Thus, trying to explore the variables that influence the incidence of left-behind children may provide suggestions for public policy making and intervention, therefore reducing the incidence of left-behind children.

# Data

## Chinese Migration Data

Migrants Population Dynamic Monitoring Survey Data (written as Chinese Migration Data) is collected by a series of surveys that are sampled with certain percentages at different regions. This data [11] is provided by the National Health Commission that previously named National Health and Family Planning Commission of People’s Republic of China.

The data is stored in CSV (comma separated values) files covering 2009 to 2014. Each row refers to a family while each column represents the answer to the questions given by a family. About 100000 rows with about 100 columns are included in a CSV file. Among these columns, some features are selected as listed in Table A. Other columns are not selected as they are not the answers to concrete questions.

**Table A. Pre-selected columns.**

|  | **Code** | **Name** | **Remark** |
| --- | --- | --- | --- |
| 1 | id | ID | ID number |
| 2 | q101b1 | gender | male:1 female:2 |
| 3 | q101d1 | from | registered province where the migrant is from |
| 4 | q101e1 | nation | nation codes are in the questionnaire |
| 5 | q101f1 | edu | the level of education |
| 6 | q101g1 | hukou | the property of the hukou, rural or urban |
| 7 | q101h1 | marriage | the state of marriage |
| 8 | q101i1 | employment | the state of employment |
| 9 | q101k1 | range | the range of this immigration |
| 10 | q203 | industry | the industry the migrant works in now |
| 11 | q204 | job | the kind of the job of the migrant |
| 12 | q205 | company | the nature of the firm |
| 13 | q206 | position | the employees role |
| 14 | q207 | income | the income of last month |
| 15 | q208 | workday | the average number of work days per week |
| 16 | q209 | workhour | the average number of work hours per day |

To study this problem, a left-behind children family should be defined so that quantitative targets can be described. The definition of the left-behind children family contains the following conditions:

• Parents or one of the parents have migrated from the place of huji to new residence

• Rural hukou for the family

• At least one child is left in the place of huji

Other definitions of left-behind children might also be reasonable. However, the definition we choose here is aligned with our data set and our goal to form the general methodology, so all the results we acquired are based on the definition and assumptions.

## Province-level Data

Province-level Data for the region is a key factor of the incidence rate of left-behind children. The Province-level Data is from the National Bureau of Statistics of the People’s Republic of China [12] as CSV files. The data are annual province data with features in 28 different fields. Dozens of features are included in each field. In our analysis, we use a data set with 1000 features as shown in Table B for the years 2013 and 2014. We further use about 100 rows of examples for each year. This data set has continuous variables for regression analysis.

**Table B. The fields in province-level data.**

|  | **Field** | **Number of Features** |  |
| --- | --- | --- | --- |
| 1 | General Survey | 59 |  |
| 2 | National Accounts | 43 |  |
| 3 | Population | 97 |  |
| 4 | Employments and Wages | 142 |  |
| 5 | Investment in Fixed Assets and Real Estate | 237 |  |
| 6 | Foreign Trade and Economic Cooperation | 13 |  |
| 7 | Energy | 45 |  |
| 8 | Finance | 48 |  |
| 9 | Price Index | 289 |  |
| 10 | People’s Living Conditions | 78 |  |
| 11 | General Survey of Cities | 69 |  |
| 12 | Resources and Environment | 179 |  |
| 13 | Agriculture | 369 |  |
| 14 | Industry | 211 |  |
| 15 | Construction | 205 |  |
| 16 | Transport and Telecommunication Services | 158 |  |
| 17 | Total Retail Sales of Consumer Goods | 1 |  |
| 18 | Wholesale and Retail Trades | 53 |  |
| 19 | Hotels and Catering Services | 41 |  |
| 20 | Tourism | 3 |  |
| 21 | Financial Intermediation | 6 |  |
| 22 | Education | 82 |  |
| 23 | Science and Technology | 90 |  |
| 24 | Public Health | 102 |  |
| 25 | Social Service | 71 |  |
| 26 | Culture | 165 |  |
| 27 | Physical Education | 20 |  |
| 28 | Public Management and Social Security | 53 |  |

# Data pre-processing

The raw Migration and Province-level Data cannot be used without processing because of missing data, different data types and unbalanced values. Hence, pre-processing is required, such as missing data deletion and imputation, normalization, discretization and bootstrapping.

For the migration data set, there are a large number of records available. Data deletion method is used to deal with the missing data problem. Afterward, there are still over 100000 records, which is enough for analysis. The records maintain the proportion for each province as in the original investigation, which is shown in Table C. Within over 100000 examples, by using the rule [13,14], K is chosen to be 18, where the inequality holds. Further, in order to ensure that there are enough examples within each interval, some extrema (or maxima since the variables are all positive) are excluded and the value X_max_ is set directly while X_min_ is 0 automatically. The only column needs to be discretized is q207(income), which is non-negative. To exclude extreme values, only the examples of q207 less than 100000 are kept.

**Table C. The sample distributions before and after deletion.**

|  | **Before Deletion** | | **After Deletion** | |
| --- | --- | --- | --- | --- |
| **Province** | **Number** | **Percentage** | **Number** | **Percentage** |
| Beijing | 100 | 0.05% | 94 | 0.05% |
| Tianjin | 184 | 0.09% | 175 | 0.10% |
| Hebei | 9245 | 4.65% | 8087 | 4.64% |
| Shanxi | 4621 | 2.32% | 3674 | 2.11% |
| Inner Mongolia | 5323 | 2.68% | 3963 | 2.28% |
| Liaoning | 2773 | 1.39% | 2514 | 1.44% |
| Jilin | 4582 | 2.30% | 3836 | 2.20% |
| Heilongjiang | 7727 | 3.89% | 6459 | 3.71% |
| Shanghai | 88 | 0.04% | 78 | 0.04% |
| Jiangsu | 6990 | 3.52% | 6319 | 3.63% |
| Zhejiang | 4833 | 2.43% | 4494 | 2.58% |
| Anhui | 16448 | 8.27% | 14355 | 8.24% |
| Fujian | 5405 | 2.72% | 4899 | 2.81% |
| Jiangxi | 9202 | 4.63% | 8565 | 4.92% |
| Shandong | 10741 | 5.40% | 9510 | 5.46% |
| Henan | 16653 | 8.38% | 15079 | 8.66% |
| Hubei | 10495 | 5.28% | 9192 | 5.28% |
| Hunan | 12468 | 6.27% | 11446 | 6.57% |
| Guangdong | 4553 | 2.29% | 4012 | 2.30% |
| Guangxi | 6797 | 3.42% | 5900 | 3.39% |
| Hainan | 1982 | 1.00% | 1652 | 0.95% |
| Chongqing | 8026 | 4.04% | 7213 | 4.14% |
| Sichuan | 16863 | 8.48% | 15140 | 8.69% |
| Guizhou | 6249 | 3.14% | 5353 | 3.07% |
| Yunnan | 4167 | 2.10% | 3822 | 2.19% |
| Tibet | 1299 | 0.65% | 1138 | 0.65% |
| Shaanxi | 6522 | 3.28% | 5445 | 3.13% |
| Gansu | 7657 | 3.85% | 6453 | 3.70% |
| Qinghai | 2458 | 1.24% | 1784 | 1.02% |
| Ningxia | 2433 | 1.22% | 2009 | 1.15% |
| Xinjiang | 1842 | 0.93% | 1470 | 0.84% |

For province-level data set, the number of the records are limited so row deletion should be avoided. Some relevant features with missing-data may be removed through column deletion. As all features are supposed equiprobably relevant, there are still relevant features contained in the data set. Therefore, only features with no missing data are included in the following work.

In this case, normalization has been applied in the Province-level Data, which means the Min-Max scaling method is applied to the data [15]. In addition, 20 times bootstrapping [16] has been done to obtain new samples with the standard deviation σ being 0.01, 0.03 and 0.05, respectively. By plotting the different samples of a feature with respect to the incidence rate, a reasonable value of variance may be determined. The figures are shown in Fig A.


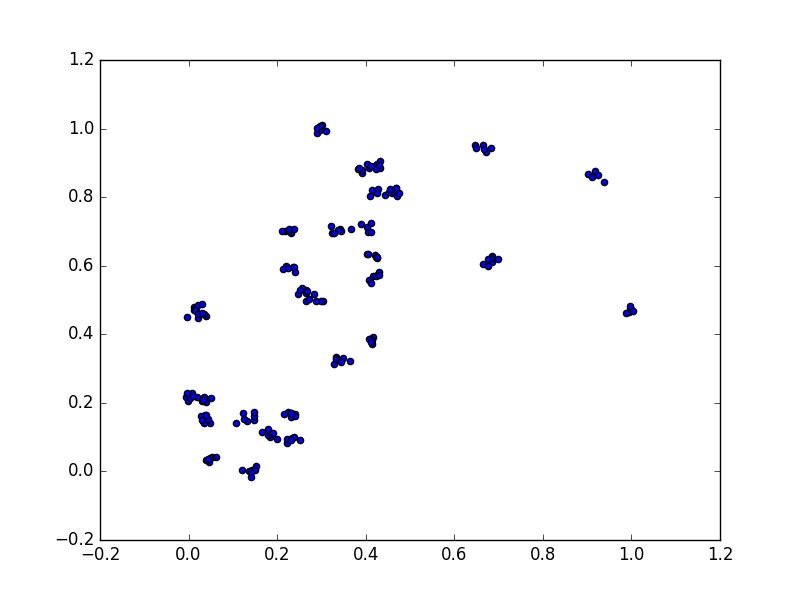

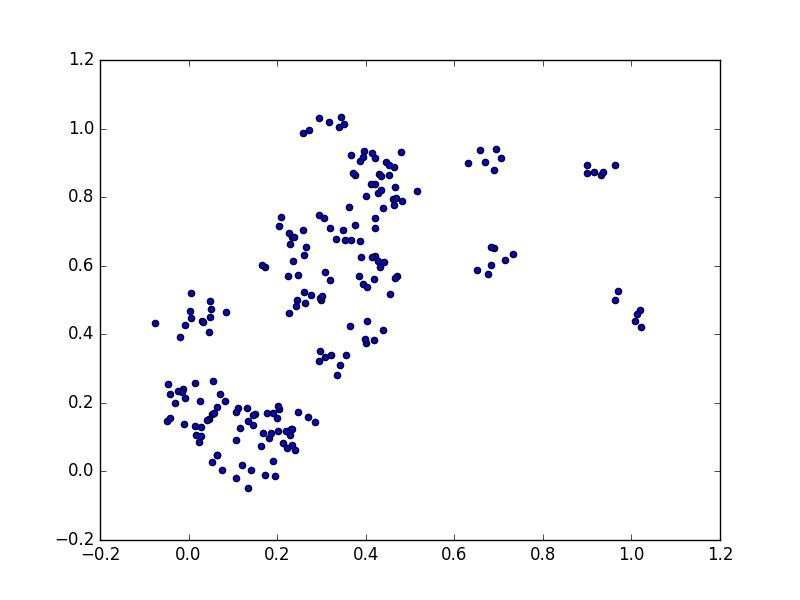

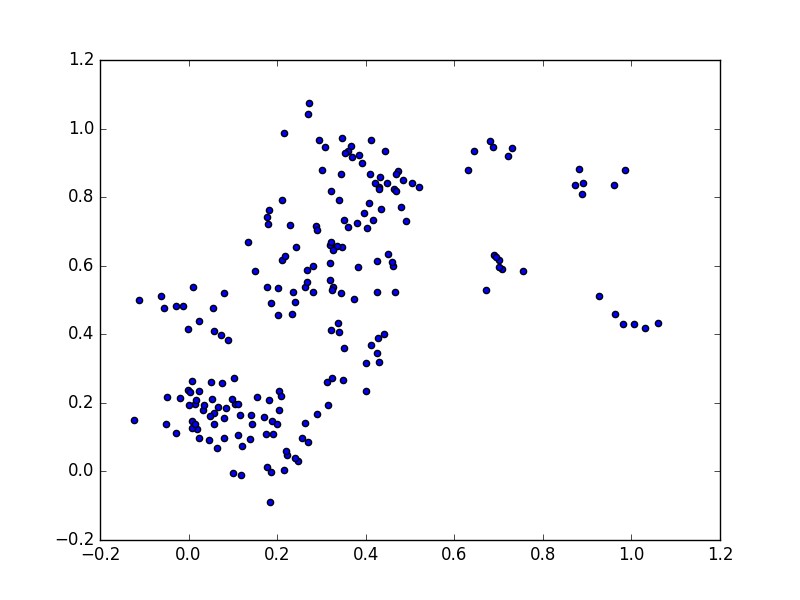


**Fig A. Distribution of standard deviations 0.01, 0.03, 0.05 from left to right.**

From the plots above, it is obvious that 0.01 standard deviation leads to an overfitting. The other two give more scattered samples. The range from 0.03 to 0.05 may be tested by involving specific models.

# Assessment models’ features

**Table D. Features of model 1.**

|  | **Feature** |
| --- | --- |
| 1 | Number of newly registered civilian vehicles |
| 2 | Number of high school graduates |
| 3 | Number of enrollment in secondary vocational schools |
| 4 | Number of national special entitled groups |
| 5 | Student-teacher ratio of primary school |

**Table E. Features of Model 2.**

|  | **Feature** |
| --- | --- |
| 1 | Real estate residential investment |
| 2 | Total wage employment in construction urban units |
| 3 | Number of middle schools |
| 4 | Student-teacher ratio of primary school |
| 5 | Number of Special Education Graduates |

**Table F. Features of Model 3.**

|  | **Feature** |
| --- | --- |
| 1 | Residential real estate sales area |
| 2 | Number of medical and health institutions discharged |
| 3 | Employees of construction enterprises |
| 4 | Number of special education enrolments |
| 5 | Number of middle school enrolments |

# References

1. Guo J, Chen L, Wang X, Liu Y, Chui CHK, He H, et al. The relationship between Internet addiction and depression among migrant children and left-behind children in China. Cyberpsychology, Behavior, and Social Networking. 2012;15(11):585–590. pages 17
2. Huang Y, Zhong XN, Li QY, Xu D, Zhang XL, Feng C, et al. Health-related quality of life of the rural-China left-behind children or adolescents and influential factors: a cross-sectional study. Health and quality of life outcomes. 2015;13(1):1. pages 17
3. Jia Z, Tian W. Loneliness of left-behind children: a cross-sectional survey in a sample of rural China. Child: care, health and development. 2010;36(6):812–817. pages 17
4. Guo J, Ren X, Wang X, Qu Z, Zhou Q, Ran C, et al. Depression among migrant and left-behind children in china in relation to the quality of parent-child and teacher-child relationships. PloS one. 2015;10(12):e0145606. pages 17
5. Liu Y, Li X, Chen L, Qu Z. Perceived positive teacher–student relationship as a protective factor for Chinese left-behind children’s emotional and behavioural adjustment. International Journal of Psychology. 2015;50(5):354–362. pages 17
6. Lu S, Lin YT, Vikse JH, Huang CC. Well-being of migrant and left-behind children in China: Education, health, parenting, and personal values. International Journal of Social Welfare. 2016;25(1):58–68. pages 17
7. Gao Y, Li LP, Kim JH, Congdon N, Lau J, Griffiths S. The impact of parental migration on health status and health behaviours among left behind adolescent school children in China. BMC public health. 2010;10(1):1. pages 17
8. Zhou C, Sylvia S, Zhang L, Luo R, Yi H, Liu C, et al. China’s left-behind children: impact of parental migration on health, nutrition, and educational outcomes. Health Affairs. 2015;34(11):1964–1971. pages 17
9. Fan F, Su L, Gill MK, Birmaher B. Emotional and behavioral problems of Chinese left-behind children: a preliminary study. Social psychiatry and psychiatric epidemiology. 2010;45(6):655–664. pages 17
10. Zhou M, Murphy R, Tao R. Effects of parents’ migration on the education of children left behind in rural China. Population and Development Review. 2014;40(2):273–292. pages 17
11. National Health and Family Planning Commission. Migrant Population Service Centre; 2018. http://www.chinaldrk.org.cn/wjw/. pages 15, 17
12. Yearbook CS, et al. National Bureau of statistics of China. China Statistical Yearbook. 2013,2014. pages 18
13. Clarke EJ, Barton BA. Entropy and MDL discretization of continuous variables for Bayesian belief networks. International Journal of Intelligent Systems. 2000;15(1):61–92. pages 19
14. Mason RD, Lind DA, Marchal WG. Statistical Techniques in Business and Economics 10 th Edition. Year: McGraw-Hill International Edition/1999; 1999. pages 19
15. Juszczak P, Tax D, Duin RP. Feature scaling in support vector data description. In: Proc. ASCI. Citeseer; 2002. p. 95–102. pages 21
16. Efron B. Bootstrap methods: another look at the jackknife. In: Breakthroughs in statistics. Springer; 1992. p. 569–593. pages 4, 5, 21
